# Proteomic and non-proteomic changes of presynaptic proteins in animal models of Alzheimer's disease: A meta-analysis 2015–2023

**DOI:** 10.1177/13872877251362212

**Published:** 2025-08-01

**Authors:** Anne Anschuetz, Karima Schwab, Charles R Harrington, Claude M Wischik, Gernot Riedel

**Affiliations:** 1 School of Medicine, Medical Sciences and Nutrition, University of Aberdeen, Aberdeen, UK; 2 TauRx Therapeutics Ltd, Aberdeen, UK

**Keywords:** aging, Alzheimer's disease, amyloid-β peptides, apolipoprotein E4, inflammation, SNARE proteins, synaptophysin, synaptosomal-associated protein 25, tau protein

## Abstract

**Background:**

Synaptic proteins are often used as markers for synaptic damage in non-clinical models of Alzheimer's disease (AD) and are known to be affected early in the disease in humans. Presynaptic protein loss has been shown to be especially strong in patients, though not all proteins and brain areas are affected equally.

**Objective:**

To investigate whether presynaptic protein loss is similarly heterogenous in non-clinical AD models, a meta-analysis was conducted in rodent models of the disease.

**Methods:**

PubMed, Embase, and Medline databases were searched for publications measuring presynaptic markers in AD rodent model brains between 2015–2023. A total of 1333 records arose from the searches, and these were screened for suitable studies. Meta-analyses on global presynaptic protein changes, protein-, area- and model-specific effects amongst others were performed on 184 studies. Studies employing proteomic approaches for protein quantification were analyzed separately from non-proteomic studies.

**Results:**

Robust and significant global loss of presynaptic proteins was observed in these animal models. The effect size varied between different model types, with the greatest effects being observed in models in which either tau was genetically modified or where amyloid-β injection was used. The greatest severity was seen in hippocampal subregions and for proteins associated with the vesicle release machinery (SNARE proteins).

**Conclusions:**

Proteomic studies confirmed that presynaptic proteins were most frequently lower in AD models relative to control, although some proteins were increased. Multiple presynaptic proteins were found to be altered in proteomic meta-analysis and some of these may constitute putative biomarkers that warrant further investigation.

## Introduction

Synaptic proteins regulate multiple processes in the synapse and are crucial for the maintenance of efficient synaptic transmission.^1^ Their loss leads to synaptic dysfunction, a key early pathological feature in neurodegeneration.^2,3^ Over the last decades, it has become clear that synapse loss is the strongest correlate for cognitive decline in Alzheimer's disease (AD).^2,4–9^ However, gross synaptic loss is not the only feature of synaptopathology in AD. Human AD patients as well as animal models of the disease also show morphological abnormalities of synapses as well as alterations in their protein machinery.^5,10–13^ Synaptic proteins have been reported to decline early in the disease process and changes correlate strongly with cognitive symptoms as well as with Braak staging.^12,13^ Proteins known to aggregate in AD, amyloid-β and tau, have been shown to mislocalize to the synapse in disease.^14–16^ These pathological proteins may directly mediate synaptic protein loss and subsequent synapse loss in AD or indirectly cause synaptic dysfunction via their effect on inflammation and other processes.^17^ A meta-analysis on human post-mortem tissue confirmed a consistent synaptic protein loss across brain regions, which was particularly strong for presynaptic proteins.^18^ The same study as well as a more recent meta-analysis revealed a distinct protein- and region-specific decline of these presynaptic proteins in AD.^18,19^ While both synapse loss and loss of presynaptic proteins have frequently been reported, it is unclear whether the complex protein- and region-specific patterns seen in humans are replicated and whether alterations in the synaptic machinery are similar in models replicating amyloid-β versus tau pathology. Since postsynaptic proteins were less affected in patients, they were excluded here.^18^ The present meta-analysis, therefore, was aimed at characterizing presynaptic protein changes in AD models to i) examine which presynaptic protein changes occur in rodents, ii) how they relate to alterations found in human AD patients, and iii) establish a relationship between the decline of presynaptic proteins as a function of underlying pathology, e.g., tau or amyloid-β. A systematic literature search from 2015 to 2023 for publications measuring proteins with presynapse function, as defined by SynGO (https://www.syngoportal.org/),^20^ in AD rodent models and wild-type controls was conducted and a meta-analysis performed on data from 184 studies, 44 of which used proteomic technologies. These proteomics studies were included and analyzed separately in an effort to report on a larger set of presynaptic proteins including less well-characterized/less frequently studied proteins. Animal models most frequently reporting presynaptic protein quantifications were genetically altered models with amyloid precursor protein (APP) and presenilin 1 (PSEN1), microtubule-associated protein tau (MAPT) mutations, viral injection of aggregation-prone genetic material, or inoculations of pathogenic extracted proteins. Amyloid-β models were most often used, including APP/PS1 mice, which carry human APP with the Swedish mutation and PSEN1 with the L166P mutation, and 5xFAD mice with three APP mutations (Swedish, Florida, London) and two PSEN1 mutations (M146L, L286V). Tau models are less represented in this dataset but included models with P301S and P301L mutations associated with frontotemporal dementia to replicate tau aggregation. The 3xTg mice were also used frequently, which carry the Swedish APP mutation, the M136V PSEN1 mutation as well as P301L tau. Common AD models have been reviewed in detail elsewhere.^21–23^ The meta-analysis findings reveal a global and reproducible presynaptic protein loss in AD models, with region- and protein-specific differences that confirm results reported for human AD patients.^11^ Crucially, the meta-analysis of proteomic studies confirmed that most presynaptic proteins decreased in AD models but also returned some unexpected presynaptic protein alterations that could be investigated in the future alongside established markers. Analyses based on both proteomic and non-proteomics studies revealed that similar protein classes were affected by AD-related pathology, with proteins of the vesicle cycle and exocytosis being the most vulnerable.

## Methods

### Search strategy

A systematic search for publications was conducted in a similar manner as reported previously.^19^ Medline, Embase, and PubMed databases from 2015 to 2023 were searched for the following keywords in abstract and title: presynaptic marker *or* presynaptic protein *or* synaptic marker *or* synaptic protein *or* proteome *and* AD *or* Alzheimer.

**Figure 1.**
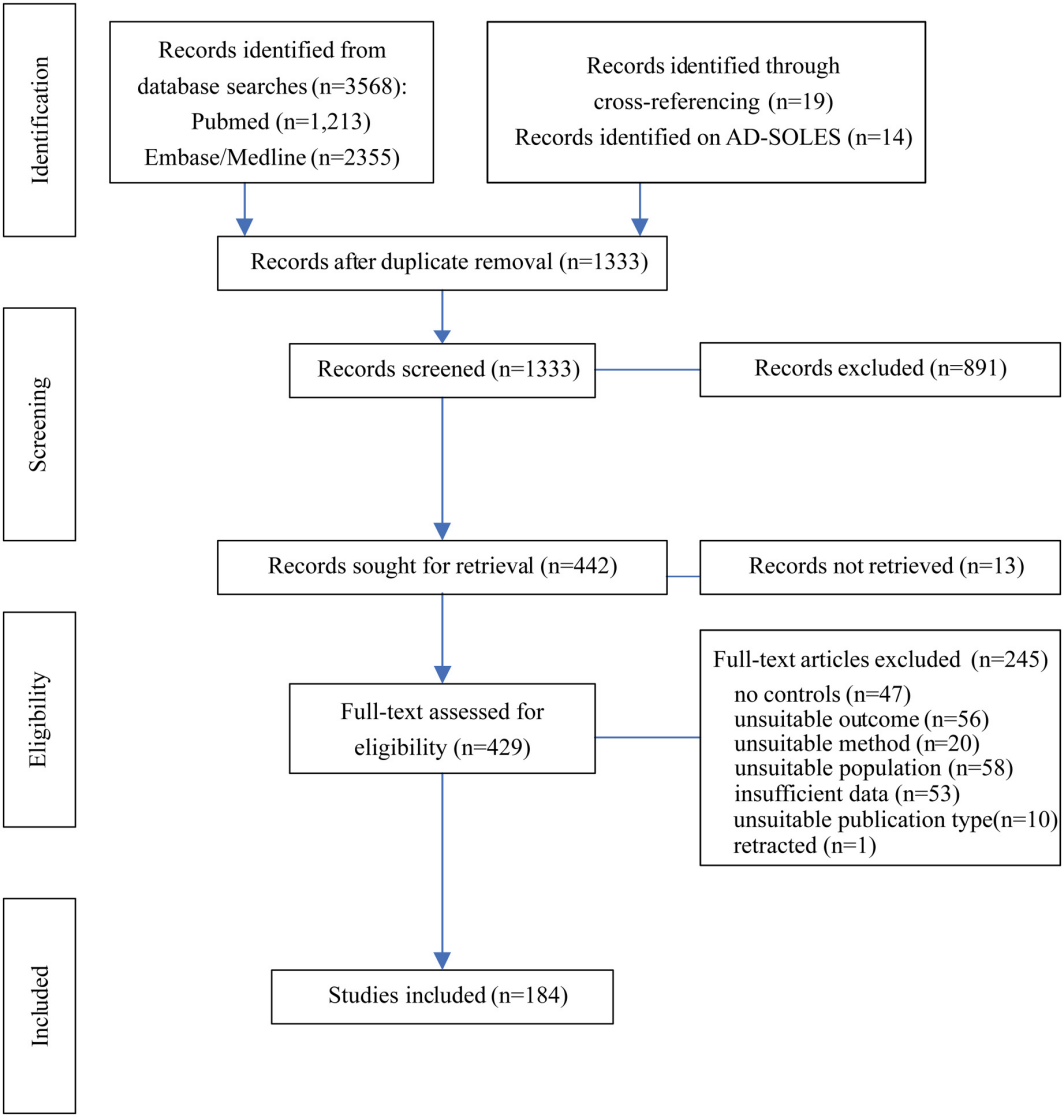
Flow chart for the systematic literature search detailing database searches and screening process. The stages of systematic search are indicated on the left, with study numbers at each stage in the middle and numbers of studies excluded on the right.

The initial search was done in February 2022, and this was updated in September 2023. Screening was conducted with the help of the systematic review tool rayyan (https://www.rayyan.ai/).^24^ After deduplication, a total of 1333 records were retrieved for title and abstract screening (Figure 1). Following this, the full text of 429 publications was assessed for inclusion while 13 records could not be retrieved in full. The criteria for inclusion required ‘AD rodent model’ and ‘wild-type (WT) or other suitable controls’, and quantification of presynaptic proteins according to the SynGO (https://www.syngoportal.org/)^20^ term “process at the presynapse” in brain tissue. Animal groups which had received a vehicle or sham treatment were also included if no untreated control groups were available. Application of the same search terms to the AD-SOLES platform (https://camarades.shinyapps.io/AD-SOLES/)^25^ returned 14 additional publications. Manual screening for cross-references of relevant articles allowed inclusion of a further 19 publications. Quantification of presynaptic proteins in other tissues but not brain, postsynaptic protein quantification or measurements of gene or mRNA expression were not included. AD-relevant models included ApoE4, SAMP8, models with pathogenic Aβ or tau Adeno-Associated Virus (AAV) injection (termed injection models), and tau and amyloid-β genetically modified (GM) models (transgenic or knock-in, e.g., 5xFAD, APP/PS1, various APP models, BRI-Aβ42, tau-P301L, tau-P301S, hTau) and triple transgenic mice (3xTg) (see Supplemental Table 1 for details). Chemically induced AD models such as scopolamine or streptozotocin injections were not considered. A total of 245 studies were excluded for failing to meet the inclusion criteria, retractions or because data provided was either insufficient for analysis or not supplied by authors upon request (Figure 1). In total, 184 publications met inclusion criteria and provided sufficient data to be included in analysis. Of these, 145 were identified as non-proteomic and 44 as proteomic studies with five using both methods and therefore included in both analyses. The number of datasets in each analysis is reported in figures and result tables.

A series of protein quantification analyses were combined under the umbrella of non-proteomic approaches. Techniques considered included immunoassays in which proteins were quantified via specific antibody-antigen interaction and visualized/quantified using chromogenic, calorimetric, chemiluminescent, radioactive or fluorescent detection tools. Such methods are immunohistochemistry (IHC); western blotting (WB); Enzyme-Linked Immunosorbent Assay (ELISA). Large scale protein quantification approaches using mass spectrometry were included in proteomic analyses.

### Data extraction

Data and study characteristics of 184 publications were extracted and used for analysis.^26–209^ Study characteristics included type of AD model, exact genotype, background strain, wild-type control strain, type of vehicle or sham treatment if applicable, age, sex, proteins measured, method used, and brain areas analyzed. Not all studies reported all these characteristic (mainly relating to age and sex). Those reports were excluded for specific subgroup analyses.

For meta-analysis, numerical data for each protein measurement was extracted from full texts/supplementary materials or from figures using the WebPlotDigitizer tool (https://automeris.io/WebPlotDigitizer/).^210^ Summary data from all available studies were extracted for each quantified protein, including AD and WT group means, standard deviation or error and sample size. When this information was not available any other statistical data that could be transformed into appropriate effect size format was extracted. A slightly different approach was followed for proteomic studies; when not enough data was available for effect size calculations, but group means, fold changes or ratios were available, these were extracted and used to generate heatmaps. They were not utilized for meta-analysis.

### Data analysis

Many studies reported multiple measurements due to analyses of several proteins, various brain areas or using multiple groups or methods. As effect size multiplicity can be challenging for meta-analyses, several strategies were applied to reduce multiplicity as described previously.^19^ In brief, when studies reported multiple measurements in multiple subregions or of different protein isoforms, effect sizes were aggregated before analysis and when different methods were used for quantification of the same protein(s) only one was selected for inclusion. Remaining multiplicity was then due to measurement of several proteins, several brain regions or different age or sex groups. In these cases, all effect sizes were included for analysis, but multiplicity was accounted for by using three-level meta-analysis in which effect sizes are clustered within individual studies.

All analyses were performed in R Studio using packages metafor,^211^ meta,^212^ dmetar^213^ and altmeta.^214^ For each meta-analysis, a random-effects multilevel meta-analysis with effect sizes clustered in studies was conducted. Heterogeneity was measured using Q-test and I^2^.^215,216^

### Non-proteomic studies

For non-proteomic studies, the standardized mean difference (SMD) adjusted for small sample sizes was used as a measure of effect size.^217^ The SMD is the difference between the WT and AD means divided by their pooled standard deviation and corrected for small sample sizes. Overall effects of AD pathology in rodents were estimated using a three-level meta-analysis on all available data and separately on data from cortex and hippocampus. We further distinguished between all models, GM models and injection models, and split according to pathology i.e., amyloid-β, tau or both (see Supplemental Table 1 for details). Subgroup analyses on different proteins, sex and age were submitted for both overall and for different types of models, and if sufficient reports were available, presynaptic protein alterations in individual AD models were also investigated. Multilevel random-effects meta-analysis was performed if five or more independent studies were accessible. Statistical significance was set at p < 0.05. Despite reduced statistical power, some relevant exploratory subgroup analyses were also performed with lower study numbers. For analysis of specific presynaptic functions, proteins were annotated with their respective functional term extracted from SynGO.

### Proteomic studies

For proteomic studies, the ratio of means (ROM) was used as index for the effect size since using the SMD would have resulted in the exclusion of numerous proteomic studies due to missing data. The ROM was calculated as ln(AD mean/WT mean). Data in multiple proteomic publications could only be used to calculate ROM but not its variance. Such data could not be used for meta-analysis but was included in heatmaps. For an overview of presynaptic protein changes, ROM values for all quantified proteins across each study were plotted in heatmaps, separated by presynaptic functions and split into the specific AD models that were examined. Remaining publications in which all necessary data for meta-analysis was available were selected for all further analysis. Separate meta-analyses were conducted for all proteins which were quantified in more than 3 independent studies and visualized in a volcano plot for each protein as -log_10_(p-value) against the ROM estimate. When data across different ages were available for a given protein, meta-regression of age (in months) was performed.

### Enrichment analysis and protein-protein interaction network for proteomic studies

Proteins that were significantly altered in the meta-analysis of proteomic studies were used for protein-protein interaction and enrichment analysis. Gene set enrichment analysis was conducted using Metascape (Version v3.5.20240901)^218^ based on ontology sources GO Biological Processes, GO Cellular Components, and GO Molecular Functions as well as biological process ontology terms within the SynGO database.^20^ The STRING database^219^ was used to generate protein-protein interaction networks which were edited in Cytoscape (Version 3.10.2).^220^

### Publication bias

To investigate publication bias, funnel plots (effect size against sample size) were visually inspected for asymmetry and a modified version of Eggers’ test, developed by Pustejovsky and Rogers^221^, was applied to reveal asymmetry (data not shown). It was assessed both globally, including all effect sizes, but also for the different types of AD models.

### Quality assessment

All studies were screened according to whether they met the full set of requirements within the essential 10 of the ARRIVE 2.0 guidelines^222^ and studies were rated on the level of detail given for each of the 10 categories. Additionally, an adapted version of the SYRCLE checklist was used to reveal risk of bias.^223^

## Results

From our systematic literature search, we included 184 studies with data from >1300 animals in this analysis (Figure 1). Of these studies, 44 reports were based on proteomic techniques including five that also validated a subset of presynaptic protein changes with other methods and were therefore included in both analyses. Models replicating amyloid-β pathology were most frequently examined in non-proteomic studies (101 in total) and consisted of 48 reports on APP/PS1 transgenic models, 16 on 5xFAD mice, 11 for various APP models, and 26 studies in which pathology was induced by injection with pathogenic amyloid-β. Eighteen studies on tau-based models were available and twenty-two publications on 3×Tg mice were found representing models with mixed tau and amyloid-β pathology. One SAMP8 and three ApoE4 models were included in our selection.

For studies using proteomic approaches, most publications were also based on amyloid-β models, including 11 studies on 5xFAD mice, 8 on APP/PS1 mice, 7 with APP animals as well as one on BRI-Aβ42 mice. Only three proteomics publications examined tau P301L or P301S transgenic mice. Two reports on 3×Tg mice as well as one recently generated mixed tau- amyloid-β model were also included in the proteomic analysis.

### Non-proteomic studies

#### Global reductions in pre-synaptic proteins in AD models

**Figure 2.**
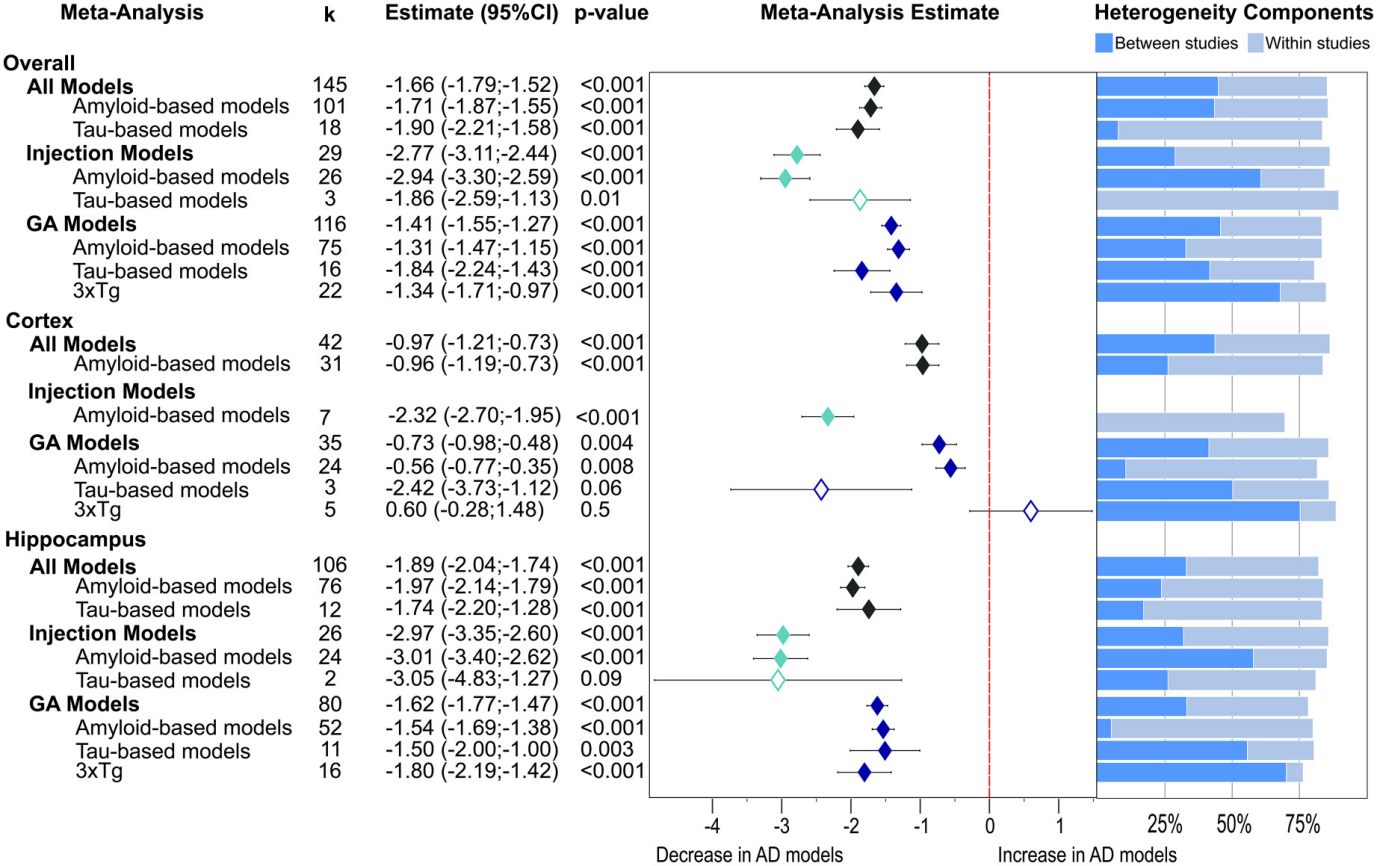
Presynaptic protein changes in AD models as found for non-proteomics studies. The results are shown as a forest plot from three-level random effects meta-analysis. Effect size estimates from each analysis are plotted with their heterogeneity components as a forest plot (left) and a stacked bar graph (right), respectively. k­ represents the number of studies contributing to each analysis. The dashed red line depicts no difference between wild-type and AD animal models. AD: Alzheimer's disease; CI: confidence interval; GM: genetically modified.

The overall effects of AD pathologies on presynaptic proteins were assessed in a multilevel meta-analysis including all effect sizes based on immunoassays (WB, ELISA, IHC) (Figure 2). Furthermore, we distinguished between effects obtained from isolated cortex and hippocampus, type of AD models (injection versus GM), and three levels of underlying pathology (amyloid-β, tau and mixed).

A global lowering of presynaptic proteins was found (Figure 2, −1.66, p < 0.001). This was due to similar effect sizes in amyloid-β models (−1.71, p < 0.001) and tau models (−1.90, p < 0.001). Intriguingly, much stronger reductions were reported for injection models (−2.77, p < 0.001) relative to GM models overall (−1.41, p < 0.001), as well as when considering amyloid-β models only (injected: −2.94, p < 0.001 versus GM: −1.31, p < 0.001). Meanwhile, the effect size was similar for both types of tau models (injected: −1.86, p = 0.01 versus GM: −1.84, p < 0.001). However, the group of tau injections was based only on three studies. Significant reductions were noted for GM models (−1.41, p < 0.001) which were equal in amyloid-β GM models (−1.31, p < 0.001) and 3xTg mice (−1.34, p < 0.001) but higher loss was reported for tau-based models (−1.84, p < 0.001).

Presynaptic protein loss was less in cortical areas (Figure 2, −0.97, p < 0.001) than in hippocampus (−1.89, p < 0.001). While this was similarly seen for amyloid-based injection (−2.32 versus −3.01, p < 0.001) and GM models (−0.56, p = 0.008 versus −1.54, p < 0.001), tau GM tissue showed greater loss in cortex (−2.42, p = 0.06 versus −1.50, p = 0.003) but the finding in cortex was based on three highly variable data sets and did not reach significance. Interestingly, five publications reported increases of presynaptic proteins in cortical tissue from 3xTg mice, but this was not significant overall (+ 0.6, p = 0.5). Again, the most severe reductions in presynaptic proteins were reported for amyloid-based injection models (cortex: −2.32; hippocampus: −3.01, p values <0.001) and for two tau viral injection studies in hippocampus (−3.05, p = 0.09) which was not significant.

Most subgroup meta-analyses showed very high heterogeneity owing to within- and between-study variability (Figure 2). The exception was for the seven amyloid-β injection models which returned moderate variability of effect sizes in cortex, which was caused solely by within-study effects.

#### Publication bias

A modified version of the Eggers’ test using all individual effect sizes of each study as well as aggregating effect sizes to one value per study and funnel plots were used to reveal any publication bias for the different subgroup analyses (data not shown). There was no evidence of funnel plot asymmetry in either overall analysis or for individual GM models analyzed separately. However, Eggers’ test was significant for injection models when all effect sizes were combined, but not when aggregating on the study level. In the funnel plot it is apparent that the asymmetry is due to missing studies with low to moderate sample sizes in which observed effects are expected to be larger than 0.

#### Presynaptic protein loss varies across AD models

**Figure 3.**
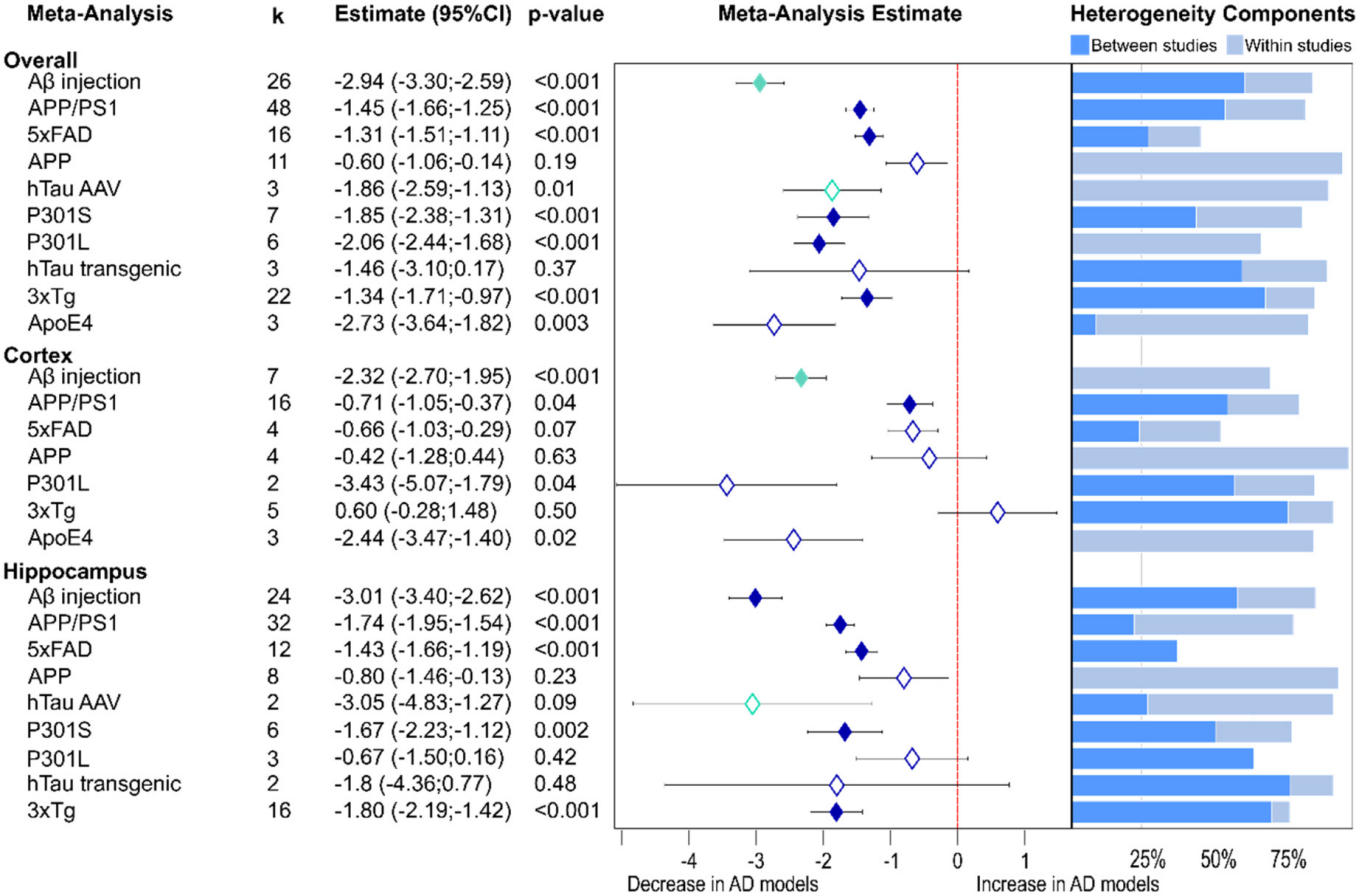
Presynaptic protein alterations in various AD models. Changes are shown as a forest plot based on non-proteomic results from three-level random effects meta-analysis. Effect size estimates from each analysis are plotted with their heterogeneity components in a forest plot (left) and stacked bar (right) graph respectively. k represents the number of studies contributing to the analysis. Dashed red line depicts no difference between wild-type and AD animal models. AAV: adeno-associated viruses; AD: Alzheimer's disease; CI: confidence interval.

Despite highly varying number of studies for each type of model (lowest 3, highest 48), presynaptic protein loss was analyzed in individual AD models. Again, the greatest reduction in synaptic proteins was seen in amyloid-β injected models (Figure 3, −2.94, see above), also observed in hippocampus (−3.01, p < 0.001). Similar lowering was observed in brain tissue from ApoE4 mice (−2.73, p = 0.003). Both P301S and P301L tau transgenic animals also showed considerable presynaptic proteins loss (P301L: −2.06, p < 0.001; P301S: −1.85, p < 0.001), as did hTau AAV models (−1.86, see above), while in a small number of other hTau transgenic models the effect was not significant. Apart from APP models, which varied little from WT controls (−0.6, p = 0.19), Aβ transgenic mice expressing multiple mutations showed moderate presynaptic protein loss (APP/PS1: −1.45, p < 0.001; 5×FAD: −1.31, p < 0.001; 3×Tg: −1.34, p < 0.001). Similar effects were seen in the different areas of interest in these individual models. Effect sizes were generally greater for all models in hippocampal tissue apart from the P301L mice where greater loss was observed in the cortex.

In the individual model meta-analysis, heterogeneity remained high in most cases with contributions from both levels. The 5xFAD model was an exception as this showed low heterogeneity in all three analyses.

#### Age-related changes in presynaptic protein loss

**Figure 4.**
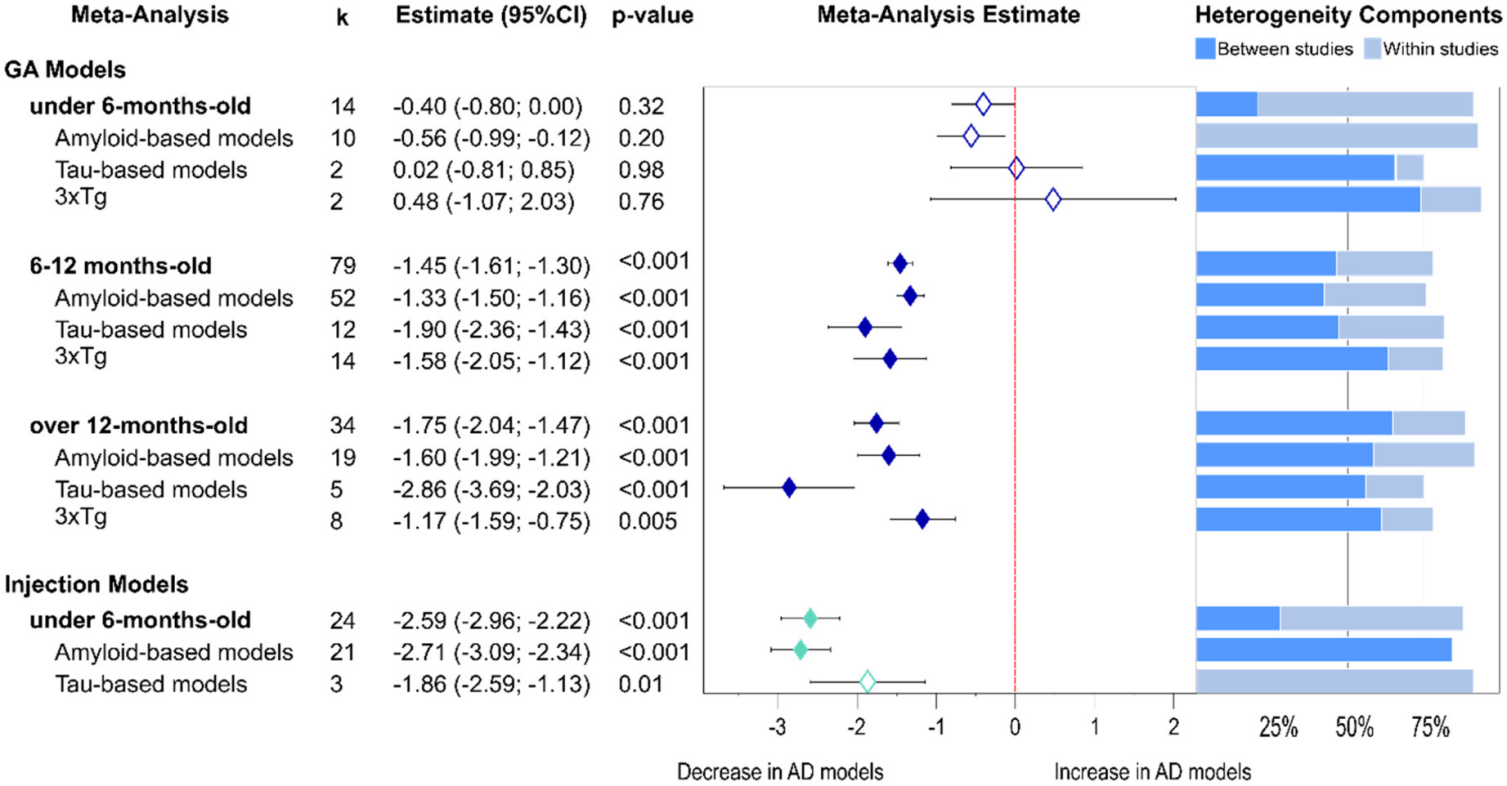
Age-related presynaptic protein changes from non-proteomics analyses. Forest plot of results from three-level random effects meta-analysis. Effect size estimates from each analysis are plotted with their heterogeneity components in a forest plot (left) and stacked bar graph (right) respectively. k represents the number of studies contributing to each analysis. Dashed red line depicts no difference between wild-type and AD animal models. k represents the number of studies contributing to the analysis. Dashed line depicts no difference between wild-type and AD animal models. AD: Alzheimer's disease; CI: confidence interval; GM: genetically modified.

We next sought to establish an age profile for the emergence and progression of presynaptic changes in the various models. Data was subdivided into three age groups: young mice under the age of six months, middle-aged mice including animals from six to twelve months of age, and old mice including all animals over twelve months (Figure 4).

Subgroup analysis was conducted for GM and injection models separately as the latter ones typically were performed on mice under 6 months of age. In young animals there was a significant decline in injection models (−2.59, p < 0.001), but no differences were observed in GM models. Furthermore, in the injection models, the effect was strongest in amyloid-β models (−2.71, p < 0.001) but also evident in the few studies available for tau models (−1.86, p = 0.011). The analyses for middle-aged and old models included only GM models, as there was only one study available for injection AD models. In these mice, there was a progressively significant presynaptic protein loss in both middle-aged (−1.45, p < 0.001) and old cohorts (−1.75, p < 0.001). The progressive decline was observed in amyloid-β GM models (−1.33 in middle aged versus −1.6 in older mice) but more prominent in tau-GM models (middle-aged: −1.90 versus old mice: −2.86). The effect of tau expression seemed stronger for synaptic protein loss than of amyloid-β. For 3xTg mice the presynaptic protein loss was significant for both ages (p values <0.005) but strongest in the middle-aged group (middle-aged: −1.58 versus old group −1.17). A more detailed analyses at the model level (Supplemental Table 2) revealed that a significant presynaptic protein loss for the 5xFAD mice was already evident in young mice (young −1.52, p < 0.001 and middle-aged: −1.23, p < 0.001) whereas, in P301L tau-transgenic mice, the decrease in presynaptic proteins became evident in old mice only (−2.83, p < 0.001).

Heterogeneity remained high in the analysis of young animals undergoing injection models (Figure 4). In GM models, it was lowest in the middle-aged group, and this was also true for amyloid-β GM models. An exception to this pattern were tau GM models, which showed only moderate heterogeneity in the old group but slightly higher variability in the middle-aged group.

#### Sex-differences in presynaptic protein alterations

Male mice were over-represented in the included publications, with 72 studies using males only, 23 using females only and 30 studies using both sexes. The remaining publications did not specify the sex used. Sex-specific differences in presynaptic protein loss are summarized in Table 1. Overall, presynaptic protein loss was stronger in male (−1.95, p < 0.001) than female mice (−1.11, p = 0.002). The same sex-specific differences were seen across models except for 5xFAD mice, where female mice had a greater presynaptic protein loss (−1.77, p = 0.012) than male mice −0.70, p = 0.002).

**Table 1.** Sex-specific changes in pre-synaptic proteins based on non-proteomics analyses.

		Meta-analysis	Heterogeneity				
	*k*	*Overall SMD [95%CI]*	*p*	*Total I^2^*	*I^2^ Level 2*	*I^2^ Level 3*	*p_Q_*
*All Models*							
Males	72	−1.95 [−2.14; −1.95]	<0.001	85.5	44.6	40.9	<0.001
Females	23	−1.11 [−1.46; −1.11]	0.002	86.8	21.7	65.1	<0.001
Amyloid-β models							
Males	57	−2.04 [−2.25; −2.04]	<0.001	84.2	30.4	53.8	<0.001
Females	12	−1.32 [−1.92; −1.32]	0.026	91	12.7	78.3	<0.001
Tau models							
Males	7	−1.78 [−2.35; −1.78]	0.002	91.7	91.7	0	<0.001
*GM Models*							
Males	46	−1.51 [−1.72; −1.51]	<0.001	82.6	40.4	42.2	<0.001
Females	21	−0.89 [−1.21; −0.89]	0.004	82.7	27.17	55.52	<0.001
Amyloid-β models							
Males	35	−1.47 [−1.70; −1.47]	<0.001	80.9	34.5	46.5	<0.001
Females	10	−0.86 [−1.32; −0.86]	0.065	84	23.4	60.5	<0.001
Tau models							
Males*	4	−1.69 [−2.69; −1.69]	0.088	93.1	93.2	0.0	<0.001
*Individual Models*							
3xTg							
Males	7	−1.58 [−2.28; −1.58]	0.025	81.9	35.8	46.1	<0.001
Females	9	−0.79 [−1.31; −0.79]	0.124	84.7	21.0	63.7	<0.001
APP/PS1							
Males	28	−1.69 [−1.96; −1.42]	<0.001	83.8	41.52	42.28	<0.001
Females	6	−0.13 [−0.60; 0.33]	0.776	80.4	31.53	48.82	<0.001
5xFAD							
Males	6	−0.70 [−0.92; −0.47]	0.002	19.8	0	19.76	0.099
Females*	3	−1.77 [−2.47; −1.07]	0.012	71.7	29.14	42.56	0.002

Meta-analysis significance indicated by *p*, Q-test significance indicated by *p*_Q_. k: Number of studies. Presynaptic protein loss tended to be higher in males than females for all analyses except 5xFAD mice where the reverse was true. Heterogeneity was high across analyses, in males mostly stemming from similar amounts of within- and between-study heterogeneity while in females, heterogeneity tended to be larger between studies than within studies;5xFAD mice again were the exception. CI: confidence interval; GM: genetically modified; SMD: standardized mean difference.

*Fewer than five independent studies available for analysis.

#### Protein changes at specific presynaptic compartments

To explore which parts of the presynapse might be particularly affected by protein alterations, we annotated proteins with the synaptic compartment in which they occur according to SynGO and conducted a separate meta-analysis. This is summarized in Table 2 and the results revealed a relatively consistent decline in synaptic proteins from all compartments and all models. An intriguing exception is the 3×Tg model which, despite the expression of multiple and additive gene mutations, presented with the weakest phenotype.

**Table 2.** Protein loss in different synaptic compartments based on non-proteomics studies.

	Meta-analysis		Heterogeneity				
Analysis	*k*	*Overall SMD [95%CI]*	*p*	*Total I^2^*	*I^2^ Level 2*	*I^2^ Level 3*	*p_Q_*
*Synaptic Vesicle*							
All Models							
Overall	135	−1.66[−1.81; −1.51]	<0.001	85.9	17.8	68.1	<0.001
Amyloid-β models	94	−1.8[−1.98; −1.62]	<0.001	86	7.0	79.0	<0.001
Tau models	18	−1.52[−1.92; −1.12]	<0.001	82.8	49.2	33.6	<0.001
Injection Models							
Overall	27	−2.78[−3.19; −2.37]	<0.001	88.9	0	88.9	<0.001
Amyloid-β models	24	−2.93[−3.34; −2.53]	<0.001	87.6	0	87.6	<0.001
Tau models*	3	−1.76[−3.87; 0.35]	0.404	95.3	0.9	94.4	0.022
GM Models							
Overall	107	−1.43[−1.58; −1.27]	<0.001	83.7	21.8	61.8	<0.001
Amyloid-β models	70	−1.44[−1.62; −1.25]	<0.001	82.7	9.92	72.8	<0.001
Tau models	15	−1.63[−2.03; −1.22]	<0.001	81.4	60.3	21.1	<0.001
3xTg	19	−1.07[−1.45; −0.68]	0.006	85.9	28.2	57.7	<0.001
*Presynaptic Membrane*							
All Models							
Overall	46	−1.76[−2.03; −1.48]	<0.001	88.3	32.2	56.2	<0.001
Amyloid-β models	28	−2.08[−2.50; −1.66]	<0.001	91.6	10.2	81.4	<0.001
Tau models	8	−2.41[−2.81; −2.02]	<0.001	82.9	82.9	0	<0.001
Injection Models							
Overall	11	−2.75[−3.17; −2.33]	<0.001	88.7	88.71	0	<0.001
Amyloid-β models	9	−3.14[−3.76; −2.53]	<0.001	86.3	21.6	64.7	<0.001
Tau models*	2	−2.24[−3.26; −1.23]	0.027	93.0	93.0	0	<0.001
GM Models							
Overall	36	−1.51[−1.83; −1.20]	<0.001	87.9	17.8	70.1	<0.001
Amyloid-β models	19	−1.56[−2.06; −1.05]	0.002	91.9	9.8	82.1	<0.001
Tau models	7	−2.47[−2.97; −1.96]	<0.001	71.9	44.3	27.6	<0.001
3xTg	10	−0.82[−1.32; −0.31]	0.104	83.6	24.4	59.2	<0.001
*Presynaptic Active Zone*							
All Models							
Overall	33	−1.07[−1.32; −0.81]	<0.001	83.2	42.9	40.3	<0.001
Amyloid-β models	18	−1.18[−1.58; −0.78]	0.00379	86.8	13.8	73.03	<0.001
Tau models	7	−1.88[−2.40; −1.37]	<0.001	86.9	86.9	0	<0.001
Injection Models							
Tau models*	2	−1.88[−3.12; −0.64]	0.130	93.9	93.9	0	<0.001
GM Models							
Overall	31	−1.00[−1.29; −0.72]	<0.001	83.5	22.2	61.3	<0.001
Amyloid-β models	17	−1.06[−1.46; −0.66]	0.008	87.1	15.9	71.2	<0.001
Tau models	6	−1.57[−2.31; −0.84]	0.032	82.5	37.0	45.5	<0.001
3xTg	8	−0.63[−1.14; −0.12]	0.216	78.4	26.3	52.2	<0.001

Meta-analysis significance indicated by *p*, Q-test significance indicated by *p*_Q_. k: Number of studies. CI: confidence interval; GM: genetically modified; SMD: standardized mean difference.

*Fewer than five independent studies available for analysis.

Most frequently analyzed were proteins located at synaptic vesicles which were overall decreased (−1.66, p < 0.001) in both injection (−2.78, p < 0.001) and GM models (−1.43, p < 0.001). Amyloid-β injection models showed the strongest loss of synaptic vesicle proteins (−2.93, p < 0.001). In GM models, however, tau overexpressing mice were more affected (−1.63, p < 0.001) than amyloid-β GM mice (−1.44, p < 0.001). Proteins located at the presynaptic membrane and presynaptic active zone also showed consistent loss across different analyses in both injection and GM models. Synaptic protein loss in 3xTg models did not attain significance (Table 2).

#### Protein functions are affected differently

**Figure 5.**
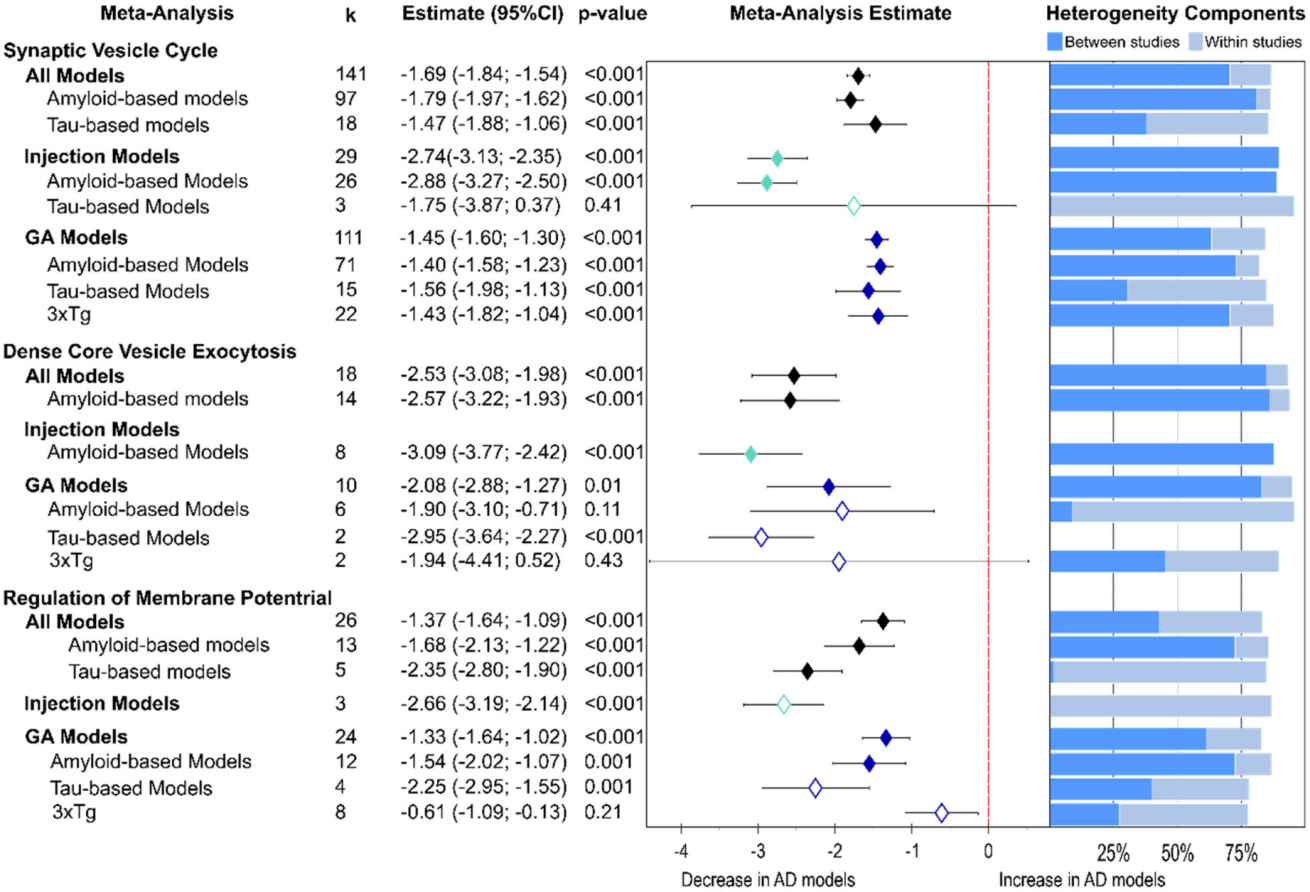
Function-specific presynaptic protein changes as found by non-proteomics analyses. Forest plot of results from three-level random effects meta-analysis. Effect size estimates from each analysis are plotted with their heterogeneity components in a forest plot (left) and stacked bar graph (right) respectively. k represents the number of studies contributing to each analysis. Dashed red line depicts no difference between wild-type and AD animal models. k represents the number of studies contributing to the analysis. AD: Alzheimer's disease; CI: confidence interval; GM: genetically modified.

Following the compartmental analysis, proteins were further classified according to their functional roles at the presynapse. Three groups were highly represented in the data analyzed here, synaptic vesicle cycle, presynaptic dense core vesicle exocytosis and presynaptic regulation of membrane potential. Almost all studies included at least one protein involved in the synaptic vesicle cycle, allowing for the most detailed subgroup analysis and returning a strong overall loss in proteins for this function (Figure 5; −1.69, p < 0.001). Amyloid and tau-based models contributed significantly to this effect size (amyloid: −1.79; tau: −1.47); injection models (−2.74, p < 0.001) presented with the biggest decline with amyloid-β injection models inducing the greatest protein loss (−2.88, p < 0.001). By contrast, in GM models the greatest protein loss was seen in tau-based models (effect size: −1.56, p < 0.001).

The most severely affected function was presynaptic dense core vesicle exocytosis (overall effect size: −2.53, p < 0.001). Again, this effect was strongest in amyloid injection (−3.09, p < 0.001) followed by tau-GM models (−2.95, p < 0.001).

For proteins involved in regulation of presynaptic membrane potential a similar pattern was observed: the strongest decline was measured in injection (−2.66, p < 0.001) and tau-GM models (−2.25, p = 0.001).

For both synaptic vesicle cycle and dense core vesicle exocytosis proteins heterogeneity was high and the result of substantial between-study variability (Figure 5). However, in the analysis of proteins involved in regulation of presynaptic membrane potential, overall heterogeneity tended to be lower and within-study differences had a larger effect.

#### Alterations in AD models are protein-specific

The ultimate non-proteomic comparison was conducted at the protein level. Synaptophysin (SYP) was by far the most frequently analyzed protein (109 studies). There was a significant overall SYP loss (Supplemental Table 3, −1.63, p < 0.001) which was similar in both amyloid (−1.78, p < 0.001) and tau models (−1.80, p < 0.001). The loss of synaptophysin was especially high in injection models (−2.79, p < 0.001) and amyloid-β models (−2.84, p < 0.001). The overall effect was smaller in GM models (−1.34, p < 0.001) and here tau-GM models had much stronger decrease of SYP (−1.77, p < 0.001) than amyloid-β models (−1.36, p < 0.001); and there was only mild loss in 3xTg mice (−0.67, p = 0.03).

Another frequently analyzed protein was Synapsin 1 (SYN1) which showed a slightly larger overall decrease than SYP (−1.74, p < 0.001), especially in GM models (−1.60, p < 0.001). For SYN1 the biggest reductions were obtained in amyloid-β models (−1.95, p < 0.001), but no significant decline was reported for 3xTg and tau models. Of note here, however, is that only two studies reported on SYN1 in tau GM mice (Supplemental Table 3).

Synaptosome Associated Protein 25 (SNAP25) was the most downregulated protein. There was a very large loss overall (−2.77, p < 0.001) as well as individually in amyloid-β and tau models. Meta-analysis on injection amyloid-β models showed strong loss in these animals compared to WT (−3.09, p < 0.001). In GM models, however, only tau mice showed SNAP25 loss with a large effect size (−2.95, p < 0.001) which in amyloid-β and 3xTg mice was not significant (Supplemental Table 3).

Proteins of the GRIN (glutamate ionotropic receptor NMDA type) and GRIA (glutamate ionotropic receptor AMPA type) family were also measured in multiple studies, allowing for subgroup meta-analyses. Loss of GRIN subunits (GRIN2A, GRIN2B, GRIN1) was most pronounced in tau GM and injection models (effect sizes < −1.88). It appears though that NMDA subunits were unaffected by multiple transgene expression in 3xTG mice (effect sizes > −0.49; all not significant). For GRIA1 subunits, GM expression of amyloid-β induced the strongest loss while in 3xTg and tau models there were no significant changes apart from a moderate increase in injection tau models. Loss in GRIA2 reached a significant level when all eight studies were considered (−1.16; p = 0.032) and in 3xTg models (−1.22, p = 0.01).

A number of other proteins were analyzed but the low number of studies involved did not allow for a discrimination between models. There was a significant loss in synaptic vesicle glycoprotein 2A (SV2A) and synaptotagmin 1 (SYT1), but syntaxin 1A (STX1A), vesicle-associated membrane protein 2 (VAMP2), cyclin dependent kinase 5 (CDK5), the solute carrier family proteins SLC17A7 (also known as VGLUT1) and SLC32A1 (also known as VGAT) showed no significant alterations (Supplemental Table 3).

Several proteins were only quantified in 3xTg mice. This included two members of the complexin family, CPLX1 and CPLX2, which both showed a large decrease in 3xTg mice as well as SYN2 which only showed a trend towards a moderate loss.

### Proteomic studies

**Figure 6.**
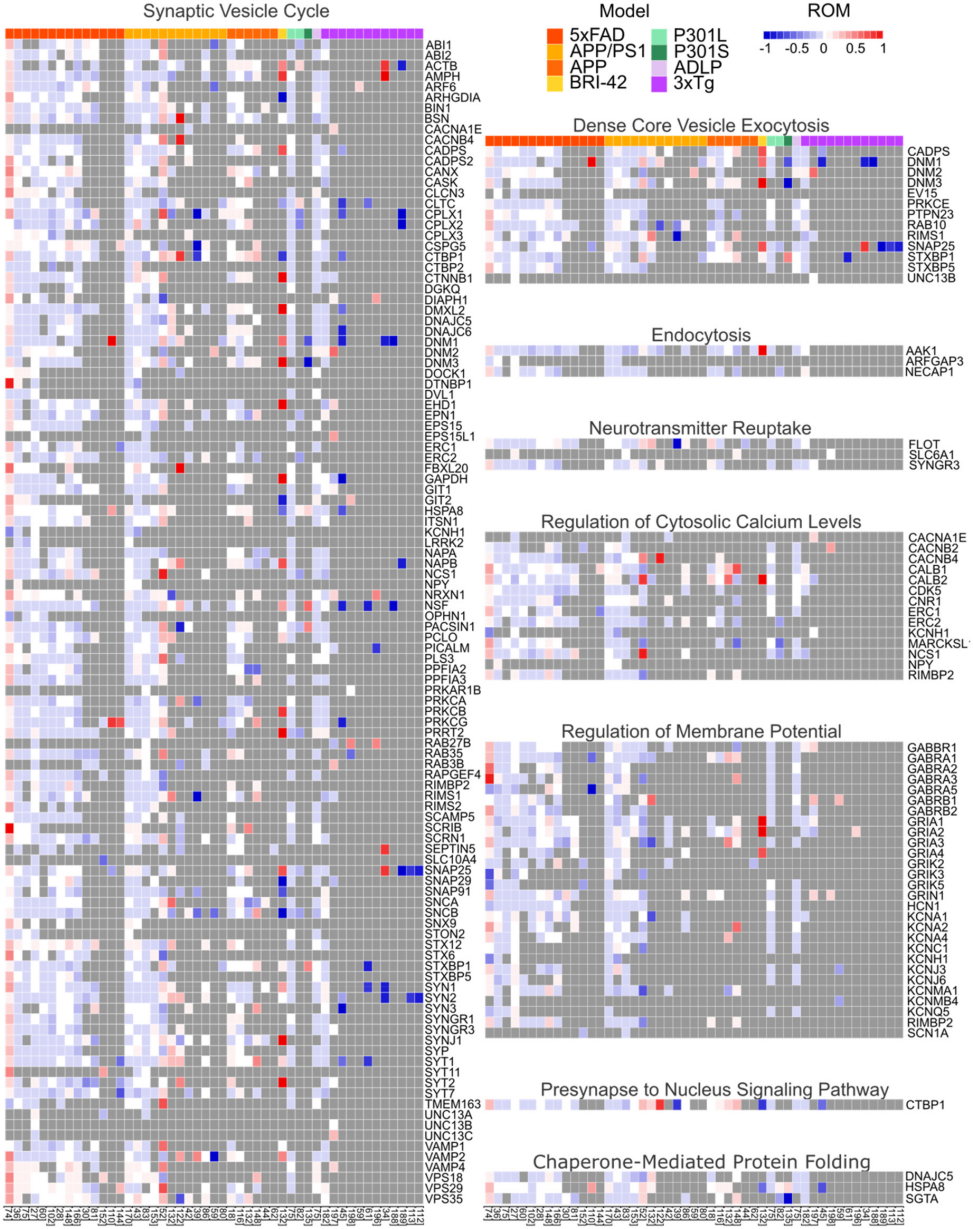
Heatmap of protein changes across presynaptic functions and ad models based on published proteomics results. ROM of all quantified proteins for the 44 available studies were categorized by function and visualized as a heatmap. The legend shows the color coding for which AD model was investigated. Grey fields indicate no available data. AD: Alzheimer's disease; ROM: ratio of means.

**Figure 7.**
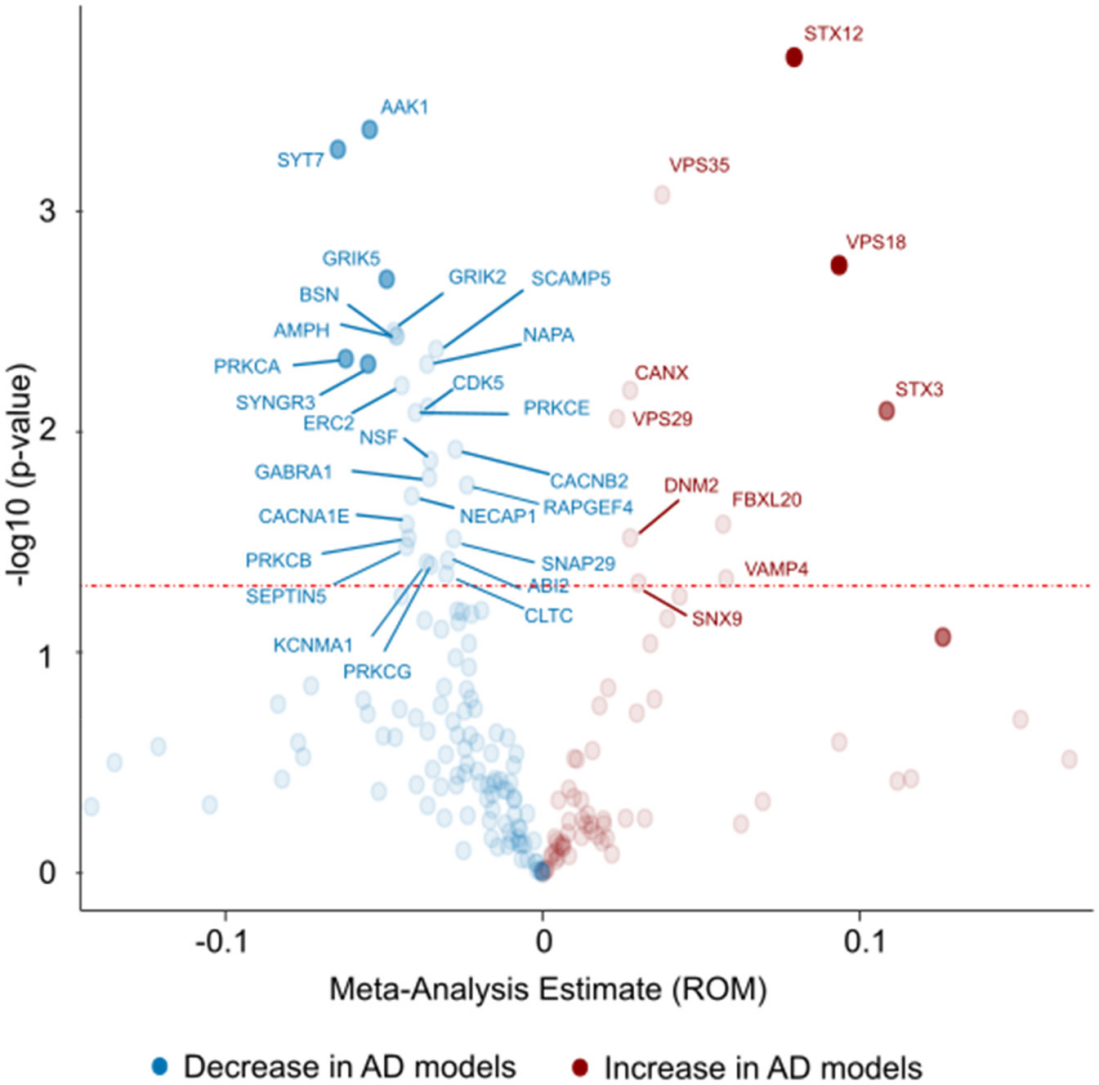
Volcano plot of significantly altered proteins as found by proteomics analyses. Twenty-nine studies were included in this meta-analysis. The meta-analysis outcome was plotted against -log10(p-value) for all proteins with sufficient data. Dashed red line indicates significance level (p = 0.05). AD: Alzheimer's disease; ROM: ratio of means.

Not all proteomic studies provided sufficient data for calculation of ratio of means (ROM) and sampling variance. Nonetheless, data from all 44 publications were still included in heatmaps used to visualize changes in the presynaptic proteome (Figure 6; note that three studies^69,85,142^ analyzed more than one model and appear multiple times in the heatmaps). The meta-analysis was conducted for 29 studies in which all required data was available, and individual proteins were analyzed if data from at least three proteomics studies was available (Figure 7).

The ROM was visualized for protein changes and used to generate heatmaps, sorted according to the different presynaptic functions and different AD models (Figure 6). Most proteins showed a small to moderate decrease across studies and different models. However, contradictory findings were also noticeable for a study on 5xFAD mice^84^ and two on APP/PS1 mice.^62,142^ Several studies on 3xTg mice reported strong decline in Synapsin 1–3,^44,55,61,122,123^ SNAP25,^122,123,190^ NSF^55,61,198^ and DNM1.^44,55,198^

When conducting a meta-analysis of individual proteins (Figure 7), a similar pattern emerged to that observed in the heatmaps. Out of the 36 significantly altered proteins, 26 were decreased in AD models (blue color in Figure 7) while only 10 were significantly increased (red color). The largest decreases were observed for SYT7, AAK1, PRKCA, SYNGR3 and GRIK2 while the largest increases were seen for STX3, VPS18 and STX12.

A total of 33 studies reported on amyloid-β models, three investigated tau-based models and 13 studies were on models with mixed pathologies; many of them only provided a subset of the data. Therefore, differences in the presynaptic proteome between models replicating different pathological hallmarks could not be investigated further.

**Figure 8.**
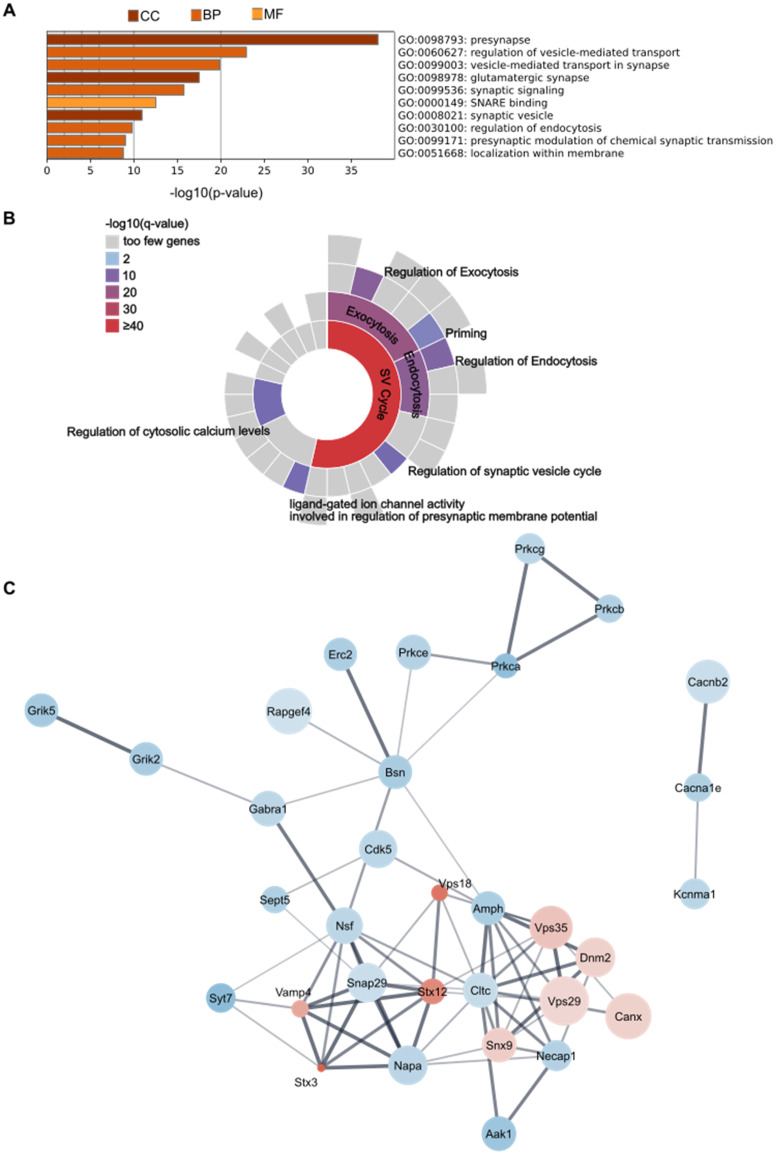
Enrichment and protein-protein-interaction analysis of significantly altered proteins from proteomics analyses. (A) GO enrichment analysis. Top ten terms using ontology sources GO Cellular Components (CC), GO Biological Processes (BP) and GO Molecular Function (MF). (B) SynGO enrichment analysis. Significantly enriched terms related to presynaptic processes are visualized in a sunburst chart. (C) Protein-Protein Interaction Network. Color of protein nodes indicates decrease (blue) or increase (red) in AD models versus WT animals, with the intensity representing strength of effect (absolute ROM value). Size of nodes is proportional to -log10(p-value). Line thickness indicates interaction score. AD: Alzheimer's disease; CC: cellular components; BP: biological processes; MF: molecular function; ROM: ratio of means; SNARE: soluble N-ethylmaleimide-sensitive-factor attachment protein receptors: SV: synaptic vesicle; WT: wildtype.

We next performed a gene set enrichment analysis to reveal the top ten enriched terms using Gene Ontology sources GO Cellular Components, GO Biological Processes, and GO Molecular Functions (Figure 8A). There was significant enrichment of proteins associated with specific cellular components including the term presynapse, which was the most highly enriched cellular component as well as the most enriched overall. Altered proteins were also associated with glutamatergic synapses and synaptic vesicles. As for biological processes, significant changes were detected for proteins involved in vesicle-mediated transport, synaptic signaling, regulation of endocytosis, modulation of chemical synaptic transmission as well as localization within membrane. The most highly enriched molecular function was SNARE binding.

Enrichment analysis for SynGO ontology terms showed high enrichment in synaptic vesicle cycle associated proteins, especially related to endo- and exocytosis (Figure 8B). Within these terms there was enrichment of proteins involved in the regulation of these processes as well as vesicle priming. Also, proteins regulating cytosolic calcium levels and ligand-gated ion channel activity that modulate membrane potential were significantly enriched.

Protein network analysis reported interactions between most altered proteins (Figure 8C). Some of the strongest interactions (indicated by the thickness of the connections) were observed between SNARE-related proteins including SNAP29, NAPA, NSF, VAMP4, STX12 and STX3. Another network with strong interactions comprised vesicular trafficking processes, such as VPS29, VPS35, DNM2, AMPH and NECAP1, which were among the most significantly increased in AD models.

Many proteomics publications investigated the AD models at multiple ages, and it was therefore possible to perform meta-regression for numerous proteins (Supplemental Figure 1). While several of these proteins were not significantly altered overall, they nonetheless showed a reliable age progression and most of them were linked to synaptic vesicle cycle and regulation of membrane potential. Fifteen of these proteins decreased over age: three were lower in AD models compared to WT already at a young age and continued to decrease further (e.g., SCN2A and PRKCA, Supplemental Figure 1A), while the remaining 12 were increased in AD models in juveniles but decreased in old cohorts >10 months (e.g., SLC1A2 and CPLX2, Supplemental Figure 1B). Nine proteins increased with advancing age: 3 were higher in AD models compared to WT from a young age and increased further (e.g., SYT11 and STX12, Supplemental Figure 1C), and 6 were lower in young AD models but increased with age (e.g., DTNBP1 and GABRA3, Supplemental Figure 1D).

### Quality assessment and risk of bias

Accurate judgements on quality of reported data using quality and risk of bias assessments were difficult to conduct as only a few studies described their methods in sufficient detail according to the ARRIVE guidelines. In particular, information on group sizes, experimental sample sizes, inclusion and exclusion criteria, randomization and blinding were lacking (Supplemental Figure 2A). Surprisingly few studies reported explicitly whether animal handlers and experimenters were blinded (and at which steps of the study), and whether studies were randomized. The lack of detail in reporting was similar between proteomics and non-proteomics studies and has not improved in nearly a decade.

Many reports were lacking details which made risk of bias judgements difficult. Due to the poor quality of reporting on details, it was not possible to determine overall study quality and risk of bias with accuracy (Supplemental Figure 2B). Blinding and randomization were only rated as “unclear”; randomization during sequence generation was reported most frequently, but the method used was not mentioned in most cases, resulting in a risk of bias rating of “moderate” for most studies. Baseline characteristics of the animals were reported in good detail; most studies achieved a rating of low to moderate risk of bias. Almost no publication provided information on whether animals were housed randomly or whether caregivers and researchers were blinded during the conduct of the experiments. If only a subset of animals was used for outcome assessment it was usually not reported whether animals were selected at random and, if so, which method was used. Frequently, sample sizes varied between different outcomes without explicit reason, e.g., exclusion criteria, so it was not clear whether there was missing outcome data. While most studies seemed to report all outcomes specified in the methods section, no study protocols were available and frequently the level of detail on outcome measures and sample sizes did not allow clear ratings due to selective outcome reporting.

## Discussion

A systematic literature search from 2015 to 2023 identified 184 publications measuring presynaptic proteins in AD rat or mouse models compared to controls. For analysis, publications were separated according to whether synaptic proteins were quantified by proteomic or immunoassay (WB, ELISA, IHC) methods. A total of 145 studies were included in the meta-analysis of non-proteomic studies, including 5 studies validating proteomic results with a non-proteomic techniques. While all 44 proteomic studies were included for visualization of protein changes using heatmaps, only 29 of these could be utilized in the meta-analysis because of missing data in the remaining 15 publications.

Our meta-analysis of studies using specific antibody-antigen interactions for protein quantification (non-proteomic) revealed significant presynaptic protein loss in AD models overall and especially in hippocampal regions. This effect was most pronounced in injection models where pathology was induced via injection of AAV (tau models only) or pathological protein (amyloid-β models only). Cerebroventricular and hippocampal amyloid-β-injection models as well as AAV tau models showed substantial neuronal loss which could contribute to the large loss of presynaptic proteins.^224–226^ Furthermore, microglia-mediated synaptic loss may play a role as inflammatory reactions like activated microglia are associated with amyloid-β or tau AAV injections.^224–227^ Lastly, the greater loss of presynaptic proteins in injection models may also be due to the invasive nature of these approaches.

The non-proteomic meta-analysis further highlighted regional differences depending on the model type. Overall, presynaptic protein loss was highest in hippocampus and had a similar magnitude in amyloid and tau-models. However, loss of presynaptic proteins in tau GM animals was greatest in cortex relative to hippocampus and amyloid-β models. While this observation is based on a low number of tau studies, it is possible that cortical neurons are more prone to tau toxicity than amyloid-β accumulation. Alternatively, the use of selected promoters may result in greater cortical expression of tau, rendering this area more vulnerable to synaptic protein loss. Indeed, some reports in GM tau models do indicate early and severe cortical tau pathology and subsequent neuronal loss.^228–230^ By contrast, 3xTg mice with mutations in amyloid-β and tau genes (i.e., APP/PS1 and P301L) did not show significant cortical presynaptic protein loss but presented with a small non-significant increase instead. Whether this is due to a gene-gene interaction remains to be explored. What has been confirmed pathologically is that 3xTg mice exhibit early cortical Aβ deposits but late onset of tau pathology which spreads from hippocampus to cortex.^231^ This late occurrence of cortical tau pathology might explain the lack of presynaptic protein loss in the cortex of 3xTg mice. Alternatively, the small non-significant increase which was also seen in under-6-month-old mice might hint at compensatory mechanisms leading to increased protein levels at early stages that cannot be maintained long-term, as has been suggested previously in humans.^232,233^

Why the presynaptic protein loss was more severe in tau-GM models than amyloid-β models for most analyses remains unclear. On one hand, the efficiency of promotors may be higher than those used for amyloid-β mice. On the other hand, a more functional explanation would suggest that tau plays a greater role in the maintenance of synaptic structures and provision of the protein machinery for the synaptic vesicle cycle, exocytosis, endocytosis and regulation of the membrane potential.^234–237^ All these elements are more severely affected in tau GM mice, while no such interactions have been reported for amyloid-β GM models. This does not explain, however, why loss of proteins was severe in amyloid-β injection models where the largest protein losses were noted. Furthermore, some proteins were specifically changed in amyloid-β (such as GRIA1) or 3xTg mice (GRIA2, CPLX1 and CPLX2) but not in tau models, suggesting some specificity for the amyloid pathways.

All AD models showed significant presynaptic protein loss as early as 6 months and the loss increased with advancing age. Since non-proteomic studies confirmed this progression primarily for synaptic proteins known to decline in AD,^18,19^ proteomic studies underlined the overall loss of presynaptic proteins and the age-related decline in AD models for many protein classes and unveiled a smaller set of presynaptic proteins that were heightened in AD models including STX (3 and 12) and VPS (18 and 29) family members.

In terms of specific protein loss, non-proteomic studies confirmed SNAP25, SYN1, SV2A, SYT1 and SYP as well as multiple receptor subunits of the GRIN (GRIN2A, GRIN2B, GRIN1) and GRIA (GRIA1, GRIA2) families as strongest decliners. Proteomic studies by contrast revealed different sets of declining proteins (AAK1, SYT7, SYNGR3) as well as few proteins that were significantly increased in AD models (STX12, VAMP4). Nonetheless, both methods identified loss in similar classes of proteins that were strongly affected in AD models including multiple SNARE and SNARE-interacting proteins, glutamate receptors and many vesicle trafficking proteins. Proteins associated with vesicle endo- and exocytosis were also highly affected. Given the more widespread analysis of proteins in the proteomic approach, it is not surprising that changes in septins, clathrin-associated proteins, calcium-signaling associated proteins or NSF and NAPA became apparent. These were typically not explored with non-proteomic methods and may represent new presynaptic markers of interest for future studies.

A comparison of the current meta-analysis of AD models with findings reported in human AD patients is difficult. First, patient cohorts are based on sporadic appearance of AD, while animal analyses contain mainly genetically modified models. Second, a clear distinction of tau and amyloid-β based pathologies is only possible for animal studies but not for human AD cohorts having mixed pathology. Third, due to a lack of data availability many subgroup analyses were only possible in humans, e.g., detailed brain regions, while others were exclusive to animal models, e.g., sex differences. Nevertheless, a great number of similarities was revealed between animals and humans. Most importantly, a global loss of presynaptic proteins as seen in humans was confirmed here.^18,19^ Further, region-specific changes reported in human patients previously were also seen in AD models. Though it was not possible to investigate brain regions as detailed as in humans due to a low number of studies quantifying proteins for example in cortical subregions, it was possible to confirm greater effect in hippocampus versus cortical regions as reported by de Wilde and colleagues.^18^ As in the human analyses, not all proteins were affected equally. SNAP25 presented with the strongest and most consistent decline in both humans and animals, while STX1A was not significantly changed in either and the frequently used marker SYP showed moderate loss in both analyses.^19^ Lastly, both systematic reviews confirmed proteins involved in vesicle exocytosis to be most vulnerable to AD pathology.^19^

### Limitations

Many studies did not provide detailed descriptions of the exact animal model used for their analysis, and this precluded an investigation into how the genetic background, promoter, expression cassette or copy number of transgene and other variations unrelated to amyloid-β or tau may affect the results. The genetic background may introduce variation in the outcome between models (i.e., amyloid-β or tau) but there will also be variation where the same model is available on different backgrounds. A study by Hurst and colleagues did find that genetic background had strong effects on the hippocampal and cortical proteome in 5xFAD transgenic mice.^70^ However, they also confirmed that proteins linked to synapses were altered by the transgenes but influenced less by the genetic background of the host species. Another important factor that might contribute to differences is the type of promoter used to generate the transgenic model. Different promoters may cause different spatial and temporal patterns of transgene expression.^238^ A lack of data on individual AD models, especially tau models, limited the extent to which model-specific differences could be investigated. Only a few models could be compared individually because few studies were available for most models. Further work is needed to confirm any model-specific effects while controlling for factors such as genetic background and promoter systems. Heterogeneity both from within- and from between-study variations was high across most analyses. Some of this variability is likely due to differences in techniques used for quantification, variations between animal models and other methodological differences. However, within-study heterogeneity is less easily explained. Measurements of different proteins across multiple brain areas or different groups of animals used (sex, age groups) may account for some variability, but the high levels of heterogeneity observed in this meta-analysis likely represent at least in part true inconsistencies for the data presented in this study.

### Conclusions

Overall presynaptic protein loss in AD models, as seen in human patients, was confirmed. While a small number of proteins increased as found by proteomic analyses, most proteins decreased in AD models compared to control, and specific protein families and functions were consistently affected, especially SNARE and vesicle-associated proteins. Proteomic studies suggested a set of new potential markers to complement established markers of presynaptic dysfunction such as STX and VPS. There were some indications for model-specific effects such as greater severity of protein loss in injection amyloid-β models and tau-GM mice but overall effects on synaptic protein loss were similar in direction.

## Supplemental Material

sj-docx-1-alz-10.1177_13872877251362212 - Supplemental material for Proteomic and non-proteomic changes of presynaptic proteins in animal models of Alzheimer's disease: A meta-analysis 2015–2023 Supplemental material, sj-docx-1-alz-10.1177_13872877251362212 for Proteomic and non-proteomic changes of presynaptic proteins in animal models of Alzheimer's disease: A meta-analysis 2015–2023 by Anne Anschuetz, Karima Schwab, Charles R Harrington, Claude M Wischik and Gernot Riedel in Journal of Alzheimer's Disease
