# Supplementary material for: Proteomic and non-proteomic changes of presynaptic proteins in animal models of Alzheimer's disease: A meta-analysis 2015–2023
Source: J Alzheimers Dis. 2025 Aug 1;107(2):452–76. doi: 10.1177/13872877251362212 (PMC12417620; doi:10.1177/13872877251362212)
Supplement: sj-docx-1-alz-10.1177_13872877251362212 - Supplemental material for Proteomic and non-proteomic changes of presynaptic proteins in animal models of Alzheimer's disease: A meta-analysis 2015–2023 [file sj-docx-1-alz-10.1177_13872877251362212.docx]

**Supplemental Material**

**Proteomic and non-proteomic changes of presynaptic proteins in animal models of Alzheimer’s disease: A meta-analysis 2015-2023**


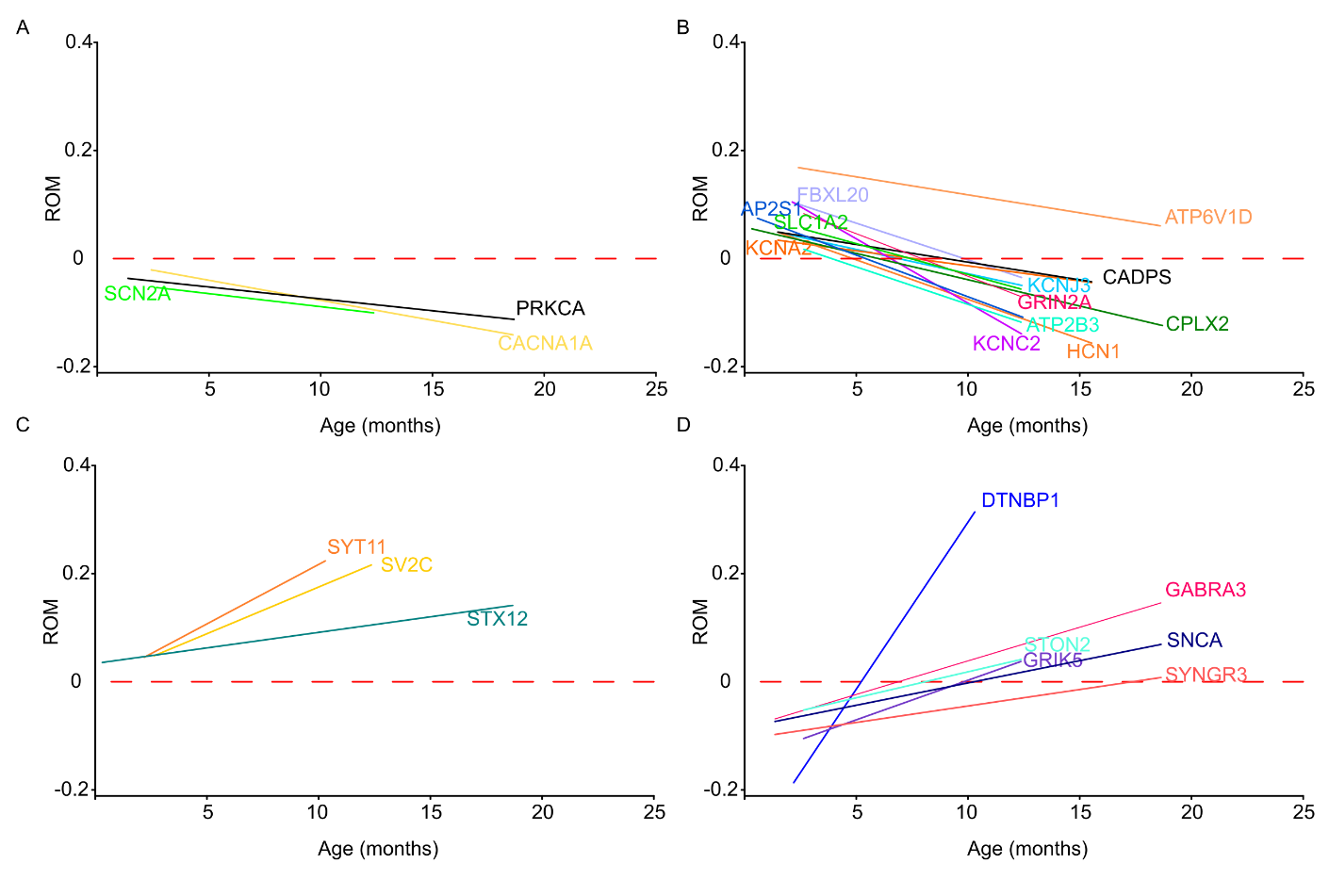


**Supplemental Figure 1.** Meta-regression plots of proteins significantly affected by age. **(**A) Proteins reduced in AD models where ROM further decreases with age. (B) Proteins increased in AD models where ROM significantly decreases with age. (C) Proteins increased in AD models where ROM further increases with age. (D) Proteins decreased in AD models where ROM increases with age. AD: Alzheimer’s disease; ROM: ratio of means.


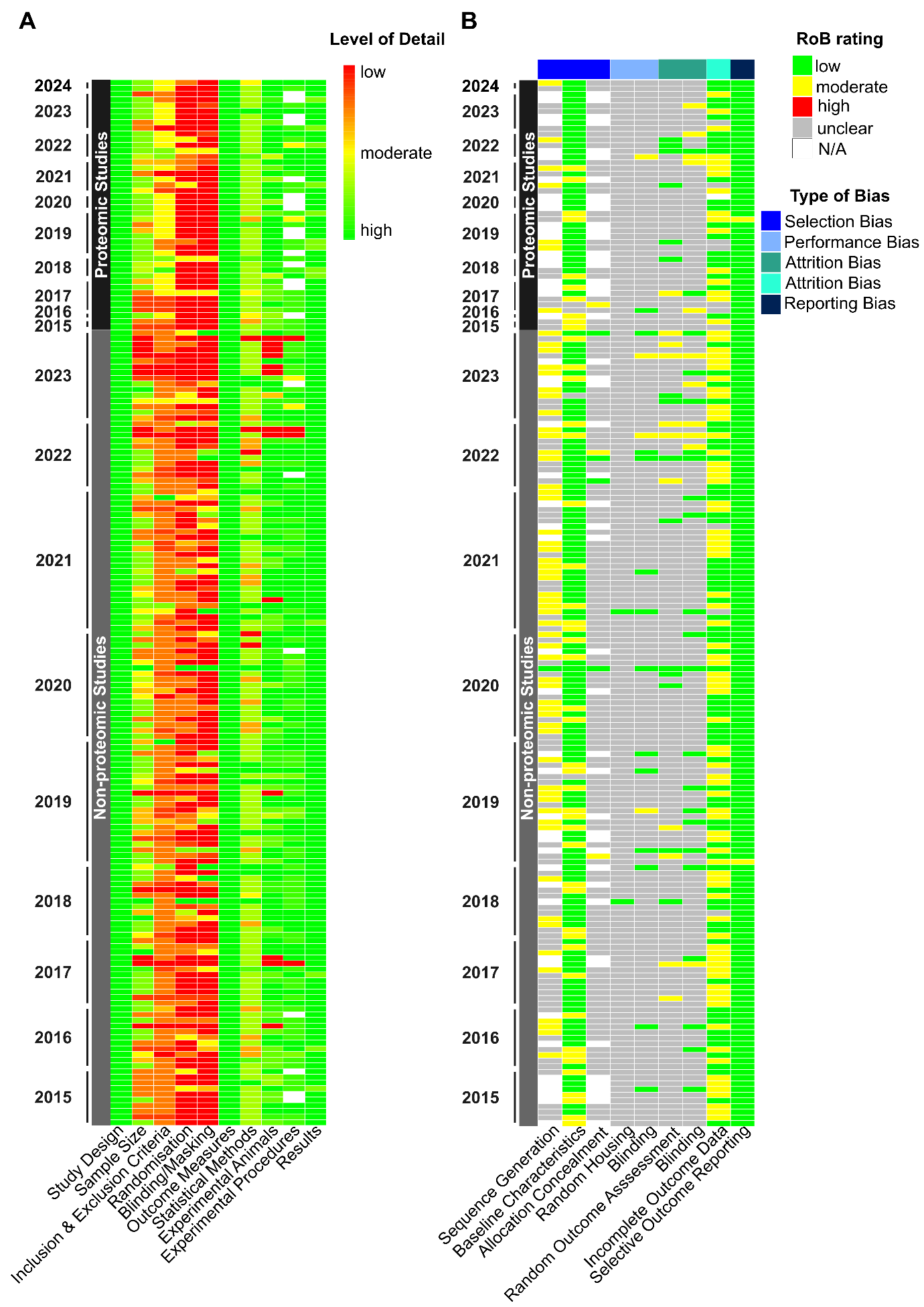
**Supplemental Figure 2.** Quality and risk of bias assessment heatmaps. (A) Level of detail reported for each of the Essential 10 ARRIVE guideline domains. (B) Risk of bias rating for 10 bias domains from the SYRCLE risk of bias tool. Each row represents one study. Publications are split into proteomic and non-proteomic studies and sorted by publication year. RoB: risk of bias

**Supplemental Table 1.** AD models used for analysis, categorized according to model type and pathology.

| Category | Pathology | Model | Citation |
| --- | --- | --- | --- |
| Injection | Amyloid-β | Amyloid-β ICV | ^1–17^ |
|  |  | Amyloid-β Intrahippocampal | ^18–23^ |
|  |  | Amyloid-β IV | ^24^ |
|  |  | Amyloid-β PFC | ^25,26^ |
|  | Tau | hTau AAV Intrahippocampal | ^27–29^ |
| GM | Amyloid-β | APP/PS1 | ^30–89^ |
|  |  | 5xFAD | ^90–119^ |
|  |  | APP transgenic (one or more pathogenic APP mutations including Swedish, Indiana, London, Florida, Iberian, Arctic) | ^70,120–131^ |
|  |  | APP KI (one or more pathogenic APP mutations including Swedish, Indiana, London, Florida, Iberian, Arctic) | ^132–135^ |
|  |  | Bri-42 | ^70^ |
|  | Tau | P301S | ^136–143^ |
|  |  | P301L | ^100,144–150^ |
|  |  | hTau transgenic (expressing full-length hTau or N- and C-terminally truncated hTau) | ^27,151,152^ |
|  | Amyloid-β and Tau | 3xTg | ^31,153–180^ |
|  |  | ADLPAPT | ^100^ |
|  | Other | *APOE4* gene-targeted replacement (carrying human *APOE* allele instead of endogenous murine *APOE* gene) | ^181^ |
|  |  | *APOE4* transgenic (carrying human *APOE4*) | ^182,183^ |
| Other |  | SAMP8 | ^184^ |

AAV: adeno-associated virus; APP: amyloid-β precursor protein; *APOE4*: apolipoprotein E4; GM: genetically modified; hTau: human tau; ICV: intracerebroventricular; IV: intravenous; KI: knock-in; PFC: prefrontal cortex; PS1: presenilin-1; SAMP8: senescence-accelerated prone 8.

**Supplemental Table 2.** Age-specific effects in various AD models.

|  | | Meta-analysis | Heterogeneity | | | | |
| --- | --- | --- | --- | --- | --- | --- | --- |
| Age (mo) | ***k*** | ***Overall SMD [95%CI]*** | ***p*** | ***Total I^2^*** | ***I^2^ Level 2*** | ***I^2^ Level 3*** | ***p_Q_*** |
| 5xFAD | | | | | | | |
| < 6 * | 3 | -1.52 [-1.81; -1.23] | <0.001 | 0 | 0 | 0 | ns |
| 6-12 | 12 | -1.23 [-1.50; -0.96] | <0.001 | 54.5 | 25.7 | 28.8 | <0.001 |
| APP/PS1 | | | | | | | |
| < 6 | 6 | -0.83 [-1.36; -0.30] | 0.118 | 79.4 | 0 | 79.4 | <0.001 |
| 6-12 | 35 | -1.40 [-1.62; -1.19] | <0.001 | 77.9 | 36.1 | 41.8 | <0.001 |
| > 12 | 12 | -2.14 [-2.72; -1.57] | <0.001 | 91.7 | 31.8 | 59.8 | <0.001 |
| APP | | | | | | | |
| 6-12 | 5 | -0.71 [-1.57; 0.15] | 0.407 | 89.4 | 37.9 | 51.5 | <0.001 |
| > 12 | 6 | -0.81 [-1.04; -0.57] | 0.001 | 78.6 | 78.6 | 0.0 | <0.001 |
| P301L tau | | | | | | | |
| 6-12 * | 4 | -1.45 [-2.61; -0.29] | 0.211 | 80.7 | 0.0 | 80.7 | 0.001 |
| > 12 * | 3 | -2.83 [-3.29; -2.36] | <0.001 | 18.9 | 0.0 | 18.9 | 0.504 |

Meta-analysis significance indicated by *p*, Q-test significance indicated by *p*_Q_. k: Number of studies; CI, confidence interval; SMD: standardized mean difference.

*Fewer than five independent studies available for analysis

**Supplemental Table 3.** Protein-specific effects.

|  | Meta-analysis | | | Heterogeneity | | | |
| --- | --- | --- | --- | --- | --- | --- | --- |
| Analysis | ***k*** | ***Overall SMD [95%CI]*** | ***p*** | ***Total I^2^*** | ***I^2^ Level 2*** | ***I^2^ Level 3*** | ***p_Q_*** |
| *Synaptophysin (SYP)* | | | | | | | |
| All Models |  |  |  |  |  |  |  |
| Overall | 110 | -1.63 [-1.80; -1.47] | <0.001 | 85.6 | 23.4 | 62.2 | <0.001 |
| Amyloid-β models | 77 | -1.78 [-1.97; -1.59] | <0.001 | 84.9 | 5.1 | 79.8 | <0.001 |
| Tau models | 15 | -1.80 [-2.31; -1.29] | <0.001 | 86.6 | 86.6 | 0 | <0.001 |
| Injection Models | | |  |  |  |  |  |
| Overall | 24 | -2.79 [-3.16; -2.41] | <0.001 | 83.5 | 0 | 83.5 | <0.001 |
| Amyloid-β models | 22 | -2.84 [-3.19; -2.48] | <0.001 | 79.9 | 0 | 79.9 | <0.001 |
| Tau models* | 2 | -2.88 [-6.64; 0.89] | 0.445 | 91.1 | 45.6 | 45.6 | 0.001 |
| GM Models | |  |  |  |  |  |  |
| Overall | 85 | -1.34 [-1.51; -1.17] | <0.001 | 84 | 28.8 | 55.3 | <0.001 |
| Amyloid-β models | 55 | -1.36 [-1.56; -1.15] | <0.001 | 82.8 | 7.2 | 75.6 | <0.001 |
| Tau models | 13 | -1.77 [-2.26; -1.27] | <0.001 | 84.5 | 84.5 | 0 | <0.001 |
| 3xTg | 14 | -0.67 [-0.99; -0.36] | 0.030 | 81.1 | 81.2 | 0 | <0.001 |
| *Synapsin 1 (SYN1)* | | | | | | | |
| All Models |  |  |  |  |  |  |  |
| Overall | 28 | -1.74 [-2.13; -1.35] | <0.001 | 87.1 | 0 | 87.1 | <0.001 |
| Amyloid-β models | 17 | -2.27 [-2.73; -1.81] | <0.001 | 84.3 | 0 | 84.3 | <0.001 |
| Tau models* | 3 | -1.40 [-2.25; -0.56] | 0.097 | 76.9 | 76.9 | 0 | 0.005 |
| Injection Models | | |  |  |  |  |  |
| Overall* | 3 | -2.53 [-4.7; -0.35] | 0.245 | 96.7 | 0 | 96.7 | <0.001 |
| Amyloid-β models* | 2 | -3.51 [-6.93; -0.09] | 0.305 | 98 | 0 | 98 | <0.001 |

| GM Models | | | |  | |  | |  | |  | |  | |  |
| --- | --- | --- | --- | --- | --- | --- | --- | --- | --- | --- | --- | --- | --- | --- |
| Overall | 25 | | | -1.60 [-1.95; -1.24] | | <0.001 | | 82.1 | | 0 | | 82.1 | | <0.001 |
| Amyloid-β models | 15 | | | -1.95 [-2.28; -1.62] | | <0.001 | | 64.5 | | 0 | | 64.5 | | <0.001 |
| Tau models* | 2 | | | -1.74 [-2.91; -0.56] | | 0.139 | | 82 | | 82 | | 0 | | <0.001 |
| 3xTg | 8 | | | -0.81 [-1.74; 0.12] | | 0.383 | | 92.3 | | 0 | | 92.3 | | <0.001 |
| *Synaptosome associated protein 25 (SNAP25)* | | | | | | | | | | | | | | |
| All Models |  | | |  | |  | |  | |  | |  | |  |
| Overall | 16 | | | -2.77 [-3.40; -2.15] | | <0.001 | | 92.3 | | 0.1 | | 92.2 | | <0.001 |
| Amyloid-β models | 12 | | | -2.89 [-3.65; -2.14] | | <0.001 | | 92.9 | | 0.1 | | 92.8 | | <0.001 |
| Tau models* | 2 | | | -2.95 [-3.64; -2.27] | | <0.001 | | 0 | | 0 | | 0 | | 0.414 |
| Injection Models | | | |  | |  | |  | |  | |  | |  |
| Amyloid-β models | 8 | | | -3.09 [-3.77; -2.42] | | <0.001 | | 86.9 | | 0 | | 86.9 | | <0.001 |
| GM Models | | | |  | |  | |  | |  | |  | |  |
| Overall | 8 | | | -2.46 [-3.52; -1.39] | | 0.021 | | 94.1 | | 47.0 | | 47.0 | | <0.001 |
| Amyloid-β models | 4 | | | -2.50 [-4.52; -0.49] | | 0.214 | | 96.1 | | 48.1 | | 48.1 | | <0.001 |
| Tau models* | 2 | | | -2.95 [-3.64; -2.27] | | <0.001 | | 0 | | 0 | | 0 | | 0.414 |
| 3xTg* | 2 | | | -1.94 [-4.41; 0.52] | | 0.430 | | 89.5 | | 44.7 | | 44.7 | | 0.002 |
| *Glutamate ionotropic receptor NMDA type subunit 2A (GRIN2A)* | | | | | | | | | | | | | | |
| All Models |  | | |  | |  | |  | |  | |  | |  |
| Overall | 16 | | | -1.58 [-2.05; -1.12] | | <0.001 | | 81.4 | | 2.2 | | 79.2 | | <0.001 |
| Amyloid-β models | 7 | | | -1.55 [-2.12; -0.98] | | 0.007 | | 77.8 | | 0 | | 77.8 | | <0.001 |
| Tau models | 5 | | | -3.18 [-3.82; -2.53] | | <0.001 | | 64.7 | | 56.1 | | 8.7 | | 0.006 |
| Injection Models | | | |  | |  | |  | |  | |  | |  |
| Tau models* | 2 | | | -4.02 [-4.80; -3.23] | | <0.001 | | 0 | | 0 | | 0 | | ns |
| GM Models | | | | |  | |  | |  | |  | |  |  |
| Overall | | 14 | | | -1.26 [-1.72; -0.79] | | 0.007 | | 80.2 | | 2.3 | | 77.9 | <0.001 |
| Amyloid-β models | | 6 | | | -1.10 [-1.54; -0.65] | | 0.014 | | 63.3 | | 7.2 | | 56.1 | 0.019 |
| Tau models* | | 4 | | | -2.98 [-3.79; -2.17] | | <0.001 | | 70.9 | | 59.9 | | 11 | 0.01 |
| 3xTg* | | 4 | | | 0.03 [-0.95; 1.01] | | 0.978 | | 82.4 | | 0 | | 82.4 | <0.001 |
| *GRIN2B* | | | | | | | | | | | | | | |
| All Models | |  | | |  | |  | |  | |  | |  |  |
| Overall | | 16 | | | -1.34 [-1.84; -0.84] | | 0.008 | | 85.9 | | 17.2 | | 68.7 | <0.001 |
| Amyloid-β models | | 8 | | | -1.09 [-1.63; -0.55] | | 0.044 | | 82.1 | | 31.2 | | 50.9 | <0.001 |
| Tau models* | | 4 | | | -3.66 [-5.15; -2.16] | | 0.015 | | 87.8 | | 45.0 | | 42.8 | <0.001 |
| Injection Models | | | | |  | |  | |  | |  | |  |  |
| Tau models* | | 2 | | | -7.45 [-10.68; -4.22] | | 0.021 | | 77.2 | | 38.6 | | 38.6 | 0.036 |
| GM Models | | | | |  | |  | |  | |  | |  |  |
| Overall | | 14 | | | -0.94 [-1.39; -0.48] | | 0.041 | | 82.2 | | 19.8 | | 62.4 | <0.001 |
| Amyloid-β models | | 7 | | | -0.76 [-1.29; -0.24] | | 0.144 | | 80.1 | | 45.2 | | 34.9 | <0.001 |
| Tau models* | | 3 | | | -2.52 [-3.92; -1.11] | | 0.07 | | 84.3 | | 24.3 | | 60.1 | 0.001 |
| 3xTg* | | 4 | | | -0.03 [-0.88; 0.81] | | 0.967 | | 76.8 | | 0 | | 76.8 | 0.004 |
| *GRIN1* | | | | | | | | | | | | | | |
| All Models | |  | | |  | |  | |  | |  | |  |  |
| Overall | | 11 | | | -0.91 [-1.29; -0.53] | | 0.017 | | 73.9 | | 18.6 | | 55.4 | <0.001 |
| Amyloid-β models | | 5 | | | -0.34 [-0.87; 0.19] | | 0.519 | | 72.7 | | 0 | | 72.7 | 0.004 |
| Tau models* | | 4 | | | -2.28 [-2.91; -1.65] | | <0.001 | | 69.2 | | 69.2 | | 0 | 0.005 |
| Injection Models | | | |  | |  | |  | |  | |  | |  |
| Tau models* | | | 2 | -3.28 [-3.97; -2.60] | | <0.001 | | 0 | | 0 | | 0 | | 0.554 |
| GM Models | | | |  | |  | |  | |  | |  | |  |
| Overall | | | 9 | -0.49 [-0.79; -0.19] | | 0.104 | | 57.7 | | 26.8 | | 30.9 | | 0.002 |
| Amyloid-β models* | | | 4 | 0.17 [-0.10; 0.44] | | 0.531 | | 0 | | 0 | | 0 | | 0.736 |
| Tau models* | | | 3 | -1.88 [-2.65; -1.10] | | 0.016 | | 72.6 | | 72.6 | | 0 | | 0.014 |
| 3xTg* | | | 2 | -0.49 [-0.82; -0.17] | | 0.129 | | 45.9 | | 45.9 | | 0 | | 0.081 |
| *Glutamate ionotropic receptor AMPA type subunit 1 (GRIA1)* | | | | | | | | | | | | | | |
| All Models | | |  |  | |  | |  | |  | |  | |  |
| Overall | | | 19 | -1.24 [-1.71; -0.76] | | 0.009 | | 86.7 | | 14.5 | | 72.3 | | <0.001 |
| Amyloid-β models | | | 8 | -2.09 [-2.76; -1.42] | | 0.002 | | 85.4 | | 10.5 | | 74.8 | | <0.001 |
| Tau models | | | 5 | -0.29 [-1.13; 0.56] | | 0.735 | | 82.7 | | 41.4 | | 41.4 | | <0.001 |
| Injection Models | | | |  | |  | |  | |  | |  | |  |
| Tau models* | | | 2 | 1.24 [0.75; 1.73] | | 0.011 | | 0 | | 0 | | 0 | | 0.601 |
| GM Models | | | |  | |  | |  | |  | |  | |  |
| Overall | | | 16 | -1.40 [-1.89; -0.91] | | 0.004 | | 85.7 | | 18.9 | | 66.8 | | <0.001 |
| Amyloid-β models | | | 7 | -1.92 [-2.68; -1.17] | | 0.011 | | 87.2 | | 13.7 | | 73.5 | | <0.001 |
| Tau models* | | | 3 | -1.41 [-2.43; -0.40] | | 0.165 | | 75.2 | | 37.6 | | 37.6 | | 0.011 |
| 3xTg | | | 6 | -0.81 [-1.74; 0.13] | | 0.387 | | 88.8 | | 15.3 | | 73.5 | | <0.001 |
| *GRIA2* | | | | | | | | | | | | | | |
| All Models | | |  |  | |  | |  | |  | |  | |  |
| Overall | | | 8 | -1.16 [-1.69; -0.62] | | 0.032 | | 78.9 | | 33.4 | | 45.5 | | <0.001 |
| Amyloid-β models* | | | 3 | -1.42 [-2.38; -0.45] | | 0.142 | | 85.3 | | 28.3 | | 57.0 | | <0.001 |
| Tau models* | | | 3 | -0.98 [-2.53; 0.58] | | 0.529 | | 90.9 | | 45.4 | | 45.4 | | 0.001 |
| Injection Models | | | |  | |  | |  | |  | |  | |  |
| Tau models* | | | 2 | 0.53 [0.07; 0.98] | | 0.251 | | 0 | | 0 | | 0 | | 0.538 |
| GM Models | | | |  | |  | |  | |  | |  | |  |
| Overall | | | 5 | -0.91 [-1.24; -0.57] | | 0.007 | | 44.5 | | 44.5 | | 0 | | 0.03 |
| Amyloid-β models* | | | 2 | -0.46 [-0.74; -0.18] | | 0.097 | | 0 | | 0 | | 0 | | 0.256 |
| 3xTg* | | | 2 | -1.22 [-1.72; -0.72] | | 0.01 | | 0 | | 0 | | 0 | | 0.429 |
| *Syntaxin 1A (STX1A)* | | | | | | | | | | | | | | |
| Overall* | | | 4 | -0.10 [-1.08; 0.88] | | 0.920 | | 88.2 | | 27.0 | | 61.2 | | <0.001 |
| *Synaptic vesicle glycoprotein 2A (SV2A)* | | | | | | | | | | | | | | |
| Overall* | | | 4 | -1.72 [-2.11; -1.34] | | <0.001 | | 41 | | 0 | | 41.0 | | 0.162 |
| *Synaptotagmin 1 (SYT1)* | | | | | | | | | | | | | | |
| Overall | | | 5 | -1.00 [-1.27; -0.72] | | <0.001 | | 0 | | 0 | | 0 | | 0.409 |
| *Vesicle-associated membrane protein 2 (VAMP2)* | | | | | | | | | | | | | | |
| Overall* | | | 3 | -0.80 [-2.25; 0.66] | | 0.583 | | 91.9 | | 45.9 | | 45.9 | | <0.001 |
| *Solute carrier family 17 member 7 (SLC17A7)* | | | | | | | | | | | | | | |
| Overall | | | 5 | -0.14 [-0.45; 0.17] | | 0.660 | | 35 | | 0 | | 35.9 | | 0.039 |
| *SLC32A1* | | | | | | | | | | | | | | |
| Overall* | | | 3 | -1.18 [-2.19; -0.17] | | 0.242 | | 81.8 | | 7.7 | | 74.1 | | <0.001 |
| *Cyclin dependent kinase 5 (CDK5)* | | | | | | | | | | | | | | |
| Overall* | | | 3 | -0.44 [-0.84; -0.04] | | 0.268 | | 58.2 | | 0 | | 58.2 | | 0.148 |
| *Complexin 1 (CPLX1)* | | | | | | | | | | | | | | |
| 3xTg* | | | 4 | -2.81 [-3.71; -1.91] | | 0.002 | | 71.7 | | 35.9 | | 35.9 | | 0.022 |
| *CPLX2* | | | | | | | | | | | | | | |
| 3xTg | | | 5 | -2.57 [-3.41; -1.73] | | 0.002 | | 74 | | 37.0 | | 37.0 | | 0.009 |
| *SYN2* | | | | | | | | | | | | | | |
| 3xTg* | | | 4 | -0.97 [-1.57; -0.38] | | 0.065 | | 63 | | 31.5 | | 31.5 | | 0.059 |

Meta-analysis significance indicated by *p*, Q-test significance indicated by *p*_Q_. k: Number of studies; CDK5: cyclin-dependent kinase 5; CI: confidence interval; CPLX: complexin; GM: genetically modified; GRIA: glutamate ionotropic receptor AMPA type subunit; GRIN: glutamate ionotropic receptor NMDA type subunit; SLC: solute carrier family; SMD: standardized mean difference; SNAP25: synaptosome associated protein 25; STX1A: syntaxin 1A; SV2A: synaptic vesicle glycoprotein 2; SYP: synaptophysin; SYN: synapsin; SYT1: synapytotagmin 1; VAMP2: vesicle-associated membrane protein 2.

*Fewer than five independent studies available for analysis

**References**

1. Ahmad A, Ali T, Park HY, et al. Neuroprotective effect of fisetin against amyloid-beta-induced cognitive/synaptic dysfunction, neuroinflammation, and neurodegeneration in adult mice. *Mol Neurobiol* 2017; 54: 2269-2285.

2. Ahmad S, Jo MH, Ikram M, et al. Deciphering the potential neuroprotective effects of luteolin against Aβ1-42-induced Alzheimer’s disease. *Int J Mol Sci* 2021; 22: 9583.

3. Ali T, Yoon GH, Shah SA, et al. Osmotin attenuates amyloid beta-induced memory impairment, tau phosphorylation and neurodegeneration in the mouse hippocampus. *Sci Rep* 2015; 5: 11708.

4. Ali, Kim MJ, Rehman SU, et al. Anthocyanin-loaded PEG-gold nanoparticles enhanced the neuroprotection of anthocyanins in an Aβ(1-42) mouse model of Alzheimer’s disease. *Mol Neurobiol* 2017; 54: 6490–6506.

5. Ali T, Kim MO. Melatonin ameliorates amyloid beta-induced memory deficits, tau hyperphosphorylation and neurodegeneration via PI3/Akt/GSk3β pathway in the mouse hippocampus. *J Pineal Res* 2015; 59: 47-59.

6. Baerends E, Soud K, Folke J, et al. Modeling the early stages of Alzheimer’s disease by administering intracerebroventricular injections of human native Aβ oligomers to rats. *Acta Neuropathol Commun* 2022; 10: 113.

7. Du Z, Song Y, Chen X, et al. Knockdown of astrocytic Grin2a aggravates β-amyloid-induced memory and cognitive deficits through regulating nerve growth factor. *Aging Cell* 2021; 20: e13437.

8. Jin S, Wang X, Xiang X, et al. Inhibition of GPR17 with cangrelor improves cognitive impairment and synaptic deficits induced by Aβ1–42 through Nrf2/HO-1 and NF-κB signaling pathway in mice. *Int Immunopharmacol* 2021; 101: 108335.

9. Khan A, Park TJ, Ikram M, et al. Antioxidative and anti-inflammatory effects of kojic acid in Aβ-induced mouse model of Alzheimer’s disease. *Mol Neurobiol* 2021; 58: 5127–5140.

10. Khan A, Park JS, Kang MH, et al. Caffeic acid, a polyphenolic micronutrient rescues mice brains against Aβ-induced neurodegeneration and memory impairment. *Antioxidants* 2023; 12: 1284

11. Kim SH, Ju IG, Kim JH, et al. Linderae radix ameliorates cognitive dysfunction by inhibiting neuroinflammation and synaptic damage in Alzheimer’s disease models. *Mol Neurobiol* 2023; 60: 7196–7207.

12. Li Q, Che H-X, Wang C-C, et al. Cerebrosides from sea cucumber improved Aβ1–42-induced cognitive deficiency in a rat model of Alzheimer’s disease. *Mol Nutr Food Res* 2019; 63: 1800707.

13. Lopez-Grancha M, Bernardelli P, Moindrot N, et al. A novel selective PKR inhibitor restores cognitive deficits and neurodegeneration in Alzheimer disease experimental models. *J Pharmacol Exp Ther* 2021; 378: 262–275.

14. Saeed K, Shah SA, Ullah R, et al. Quinovic acid impedes cholesterol dyshomeostasis, oxidative stress, and neurodegeneration in an amyloid-β-induced mouse model. *Oxid Med Cell Longev* 2020; 2020: 9523758.

15. Torrisi SA, Geraci F, Tropea MR, et al. Fluoxetine and vortioxetine reverse depressive-like phenotype and memory deficits induced by Aβ1-42 oligomers in mice: A key role of transforming growth factor-β1. *Front Pharmacol* 2019; 10: 693.

16. Watremez W, Jackson J, Almari B, et al. Stabilized low-n amyloid-β oligomers induce robust novel object recognition deficits associated with inflammatory, synaptic, and GABAergic dysfunction in the rat. *J Alzheimers Dis* 2018; 62: 213–226.

17. Wu X, Lv Y-G, Du Y-F, et al. Neuroprotective effects of INT-777 against Aβ1–42-induced cognitive impairment, neuroinflammation, apoptosis, and synaptic dysfunction in mice. *Brain Behav Immun* 2018; 73: 533–545.

18. Chang K-W, Zong H-F, Rizvi MY, et al. Modulation of the MAPKs pathways affects Aβ-induced cognitive deficits in Alzheimer’s disease via activation of α7nAChR. *Neurobiol Learn Mem* 2020; 168: 107154.

19. Cui G-H, Shao S-J, Yang J-J, et al. Designer self-assemble peptides maximize the therapeutic benefits of neural stem cell transplantation for Alzheimer’s disease via enhancing neuron differentiation and paracrine action. *Mol Neurobiol* 2016; 53: 1108–1123.

20. Ghumatkar P, Peshattiwar V, Patil S, et al. The effect of phloretin on synaptic proteins and adult hippocampal neurogenesis in Aβ (1-42)-injected male Wistar rats. *J Pharm Pharmacol* 2018; 70: 1022–1030.

21. Hidisoglu E, Kantar D, Ozdemir S, et al. Cognitive dysfunctions and spontaneous EEG alterations induced by hippocampal amyloid pathology in rats. *Adv Med Sci* 2022; 67: 328--337.

22. Lee Y-S, Lai D-M, Huang H-J, et al. Prebiotic lactulose ameliorates the cognitive deficit in Alzheimer’s disease mouse model through macroautophagy and chaperone-mediated autophagy pathways. *J Agric Food Chem* 2021; 69: 2422–2437.

23. Shi J, Li Y, Zhang Y, et al. Baicalein ameliorates Aβ-induced memory deficits and neuronal atrophy via inhibition of PDE2 and PDE4. *Front Pharmacol* 2021; 12: 794458.

24. Han Y, Chen L, Guo Y, et al. Class I HDAC inhibitor improves synaptic proteins and repairs cytoskeleton through regulating synapse-related genes in vitro and in vivo. *Front Aging Neurosci* 2020; 12: 619866.

25. Shi Y, Lv J, Chen L, et al. Phosphodiesterase-4D knockdown in the prefrontal cortex alleviates memory deficits and synaptic failure in mouse model of Alzheimer’s disease. *Front Aging Neurosci* 2021; 13: 722580.

26. Yan Y, Gao S, Avasthi S, et al. Protective effects of phosphodiesterase 2 inhibitor against Aβ1-42 induced neuronal toxicity. *Neuropharmacology* 2022; 213: 109128.

27. Li X, Hong X, Wang Y, et al. Tau accumulation triggers STAT1‐dependent memory deficits by suppressing NMDA receptor expression. *EMBO Rep* 2019; 20: e47202.

28. Wan H-L, Hong X-Y, Zhao Z-H, et al. STAT3 ameliorates cognitive deficits via regulation of NMDAR expression in an Alzheimer’s disease animal model. *Theranostics* 2021; 11: 5511–5524.

29. Zhang Q, Xia Y, Luo H, et al. Codonopsis pilosula polysaccharide attenuates tau hyperphosphorylation and cognitive impairments in hTau infected mice. *Front Mol Neurosci* 2018; 11: 437.

30. Arora, Kanekiyo T., Singh J. Functionalized nanoparticles for brain targeted BDNF gene therapy to rescue Alzheimer’s disease pathology in transgenic mouse model. *Int J Biol Macromol* 2022; 208: 901-911.

31. Huang X, Yang J, Huang X, et al. Tetramethylpyrazine improves cognitive impairment and modifies the hippocampal proteome in two mouse models of Alzheimer’s disease. *Front Cell Dev Biol* 2021; 9: 632843.

32. Cao K, Xiang J, Dong Y-T, et al. Exposure to fluoride aggravates the impairment in learning and memory and neuropathological lesions in mice carrying the APP/PS1 double-transgenic mutation. *Alzheimers Res Ther* 2019; 11: 35.

33. Chacón PJ, del Marco Á, Arévalo Á, et al. Cerebellin 4, a synaptic protein, enhances inhibitory activity and resistance of neurons to amyloid-β toxicity. *Neurobiol Aging* 2015; 36: 1057–1071.

34. Clement A, Madsen MJ, Kastaniegaard K, et al. Chronic stress induces hippocampal mitochondrial damage in APPPS1 model mice and wildtype littermates. *J Alzheimers Dis* 2022; 87: 259–272.

35. Deng L, Gupta VK, Wu Y, et al. Mouse model of Alzheimer’s disease demonstrates differential effects of early disease pathology on various brain regions. *Proteomics* 2021; 21: e2000213.

36. de Veij Mestdagh C.F., Koopmans F, Breiter J.C, et al. The hibernation-derived compound SUL-138 shifts the mitochondrial proteome towards fatty acid metabolism and prevents cognitive decline and amyloid plaque formation in an Alzheimer’s disease mouse model. *Alzheimers Res Ther* 2022; 14: 183.

37. Du Y, Qu J, Zhang W, et al. Morin reverses neuropathological and cognitive impairments in APPswe/PS1dE9 mice by targeting multiple pathogenic mechanisms. *Neuropharmacol* 2016; 108: 1–13.

38. Fu X-X, Wei B, Cao H-M, et al. Telmisartan alleviates Alzheimer’s disease-related neuropathologies and cognitive impairments. *J Alzheimers Dis*2023; 94: 919-933.

39. Gu X-H, Xu L-J, Liu Z-Q, et al. The flavonoid baicalein rescues synaptic plasticity and memory deficits in a mouse model of Alzheimer’s disease. *Behav Brain Res* 2016; 311: 309-321.

40. Hamezah HS, Durani LW, Yanagisawa D, et al. Modulation of proteome profile in AβPP/PS1 mice hippocampus, medial prefrontal cortex, and striatum by palm oil derived tocotrienol-rich fraction. *J Alzheimers Dis* 2019; 72: 229–246.

41. Ji S, Wu Y, Zhu R, et al. Novel phenylethanoid glycosides improve hippocampal synaptic plasticity via the cyclic adenosine monophosphate-CREB-brain-derived neurotrophic growth factor pathway in APP/PS1 transgenic mice. *Gerontology* 2023; 69: 1065–1075.

42. Jin G, Zhu L, Liu P, et al. Xanthoceraside prevented synaptic loss and reversed learning-memory deficits in APP/PS1 transgenic mice. *J Physio Sci* 2019; 69: 477-488.

43. Kelliny S, Lin L, Deng I, et al. A new approach to model sporadic Alzheimer’s disease by intracerebroventricular streptozotocin injection in APP/PS1 mice. *Mol Neurobiol* 2021; 58: 3692–3711.

44. King C and Robinson R. Evaluating combined precursor isotopic labeling and isobaric tagging performance on orbitraps to study the peripheral proteome of Alzheimer’s disease. *Anal Chem* 2020; 92: 2911–2916.

45. Kotah JM, Kater M, y S. J., et al. Early-life stress and amyloidosis in mice share pathogenic pathways involving synaptic mitochondria and lipid metabolism. *Alzheimers Dement* 2024; 20: 1637--1655.

46. Kusakari S, Nawa M, Sudo K, et al. Calmodulin-like skin protein protects against spatial learning impairment in a mouse model of Alzheimer disease. *J Neurochem* 2018; 144: 218–233.

47. Kuwar R, Rolfe A, Di L, et al. A novel inhibitor targeting NLRP3 inflammasome reduces neuropathology and improves cognitive function in Alzheimer’s disease transgenic mice. *J Alzheimers Dis* 2021; 82: 1769–1783.

48. Lachén-Montes M, González-Morales A, de Morentin XM, et al. An early dysregulation of FAK and MEK/ERK signaling pathways precedes the β-amyloid deposition in the olfactory bulb of APP/PS1 mouse model of Alzheimer’s disease. *J Proteomics* 2016; 148: 149–158.

49. Lee C-C, Chang C-P, Lin C-J, et al. Adenosine augmentation evoked by an ENT1 inhibitor improves memory impairment and neuronal plasticity in the APP/PS1 mouse model of Alzheimer’s disease. *Mol Neurobiol* 2018; 55: 8936–8952.

50. Li B, Liu J, Gu G, et al. Impact of neural stem cell‐derived extracellular vesicles on mitochondrial dysfunction, sirtuin 1 level, and synaptic deficits in Alzheimer’s disease. *J Neurochem* 2020; 154: 502–518.

51. Li T, Martin E, Abada Y, et al. Effects of chronic masitinib treatment in APPswe/PSEN1dE9 transgenic mice modeling Alzheimer’s disease. *J Alzheimers Dis* 2020; 76: 1339–1345.

52. Li X, He Z, Wang C, et al. Effect of herbal medicinal compounds on Alzheimer’s disease pathology in APP/PS1 transgenic mouse model. *Nat Prod Commun 2020; 15: 1934578X20948986.*

53. Li Z, Zhang X-B, Gu J-H, et al. Breviscapine exerts neuroprotective effects through multiple mechanisms in APP/PS1 transgenic mice. *Mol Cell Biochem* 2020; 468: 1–11.

54. Liang X, Yao Y, Lin Y, et al. Panaxadiol inhibits synaptic dysfunction in Alzheimer’s disease and targets the Fyn protein in APP/PS1 mice and APP-SH-SY5Y cells. *Life Sci* 2019; 221: 35–46.

55. Liu B, Hinshaw RG, Le KX, et al. Space-like 56Fe irradiation manifests mild, early sex-specific behavioral and neuropathological changes in wildtype and Alzheimer’s-like transgenic mice. *Sci Rep* 2019; 9: 12118.

56. Liu Y, Hu P-P, Zhai S, et al. Aquaporin 4 deficiency eliminates the beneficial effects of voluntary exercise in a mouse model of Alzheimer’s disease. *Neural Regen Res 2022; 17: 2079–2088.*

57. Lopes CR, Silva JS, Santos J, et al. Downregulation of sirtuin 1 does not account for the impaired long-term potentiation in the prefrontal cortex of female APPswe/PS1dE9 mice modelling Alzheimer’s disease. *Int J Mol Sci* 2023; 24: 6968.

58. Mahaman YAR, Huang F, Salissou MTM, et al. Ferulic acid improves synaptic plasticity and cognitive impairments by alleviating the PP2B/DARPP-32/PP1 axis-mediated STEP increase and Aβ burden in Alzheimer’s disease. *Neurother* 2023; 20: 1081–1108.

59. Mampay M, Velasco‐Estevez M, Rolle SO, et al. Spatiotemporal immunolocalisation of REST in the brain of healthy ageing and Alzheimer’s disease rats. *FEBS Open Bio* 2020; 11: 146–163.

60. Mengr A, Hrubá L, Exnerová A, et al. Palmitoylated prolactin-releasing peptide reduced Aβ plaques and microgliosis in the cerebellum: APP/PS1 mice study. *Curr Alzheimers Res* 2021; 18: 607–622.

61. Ostapchenko VG, Chen M, Guzman MS, et al. The transient receptor potential melastatin 2 (TRPM2) channel contributes to β-amyloid oligomer-related neurotoxicity and memory impairment. *J Neurosci* 2015; 35: 15157–15169.

62. Petukhova EO, Mukhamedshina YO, Salafutdinov II, et al. Effects of transplanted umbilical cord blood mononuclear cells overexpressing GDNF on spatial memory and hippocampal synaptic proteins in a mouse model of Alzheimer’s disease. *J Alzheimers Dis* 2019; 69: 443–453.

63. Qi F, Zuo Z, Hu K, et al. VEGF-A in serum protects against memory impairment in APP/ PS1 transgenic mice by blocking neutrophil infiltration. *Mol Psychiatry* 2023; 28: 4374-4389.

64. Rao SK, Ross JM, Harrison FE, et al. Differential proteomic and behavioral effects of long-term voluntary exercise in wild-type and APP-overexpressing transgenics. *Neurobiol Dis* 2015; 78: 45–55.

65. Rodrigues B dos S, Kanekiyo T, Singh J. Nerve growth factor gene delivery across the blood–brain barrier to reduce beta amyloid accumulation in AD mice. *Mol Pharmaceutics* 2020; 17: 2054–2063.

66. Rudobeck E, Bellone JA, Szücs A, et al. Low-dose proton radiation effects in a transgenic mouse model of Alzheimer’s disease – Implications for space travel. *PLoS One* 2017; 12: e0186168.

67. Salazar SV, Gallardo C, Kaufman AC, et al. Conditional deletion of Prnp rescues behavioral and synaptic deficits after disease onset in transgenic Alzheimer’s disease. *J Neurosci* 2017; 37: 9207-9221.

68. Salazar SV, Cox TO, Lee S, et al. Alzheimer’s disease risk factor Pyk2 mediates amyloid-β-induced synaptic dysfunction and loss. *J Neurosci* 2019; 39: 758–772.

69. Sanchez-Varo R, Sanchez-Mejias E, Fernandez-Valenzuela JJ, et al. Plaque-associated oligomeric amyloid-beta drives early synaptotoxicity in APP/PS1 mice hippocampus: ultrastructural pathology analysis. *Front Neurosci* 2021; 15: 752594.

70. Savas JN, Wang Y-Z, DeNardo LA, et al. Amyloid accumulation drives proteome-wide alterations in mouse models of Alzheimer’s disease-like pathology. *Cell Rep* 2017; 21: 2614–2627.

71. Shi Q, Chowdhury S, Ma R, et al. Complement C3 deficiency protects against neurodegeneration in aged plaque-rich APP/PS1 mice. *Sci Transl Med* 2017; 9: eaaf6295.

72. Stuart KE, King AE, Fernandez-Martos CM, et al. Mid-life environmental enrichment increases synaptic density in CA1 in a mouse model of Aβ-associated pathology and positively influences synaptic and cognitive health in healthy ageing. *J Comp Neurol* 2017; 525: 1797–1810.

73. Sun T, Shi Q, Zhang Y, et al. Focused ultrasound with anti-pGlu3 Aβ enhances efficacy in Alzheimer’s disease-like mice via recruitment of peripheral immune cells. *J Control Release* 2021; 336: 443–456.

74. Szögi T, Schuster I, Borbély E, et al. Effects of the pentapeptide P33 on memory and synaptic plasticity in APP/PS1 transgenic mice: A novel mechanism presenting the protein Fe65 as a target. *Int J Mol Sci* 2019; 20: 3050.

75. Tai J, Liu W, Li Y, et al. Neuroprotective effects of a triple GLP-1/GIP/glucagon receptor agonist in the APP/PS1 transgenic mouse model of Alzheimer’s disease. *Brain Res* 2018; 1678: 64–74.

76. Thygesen C, Ilkjær L, Kempf SJ, et al. Diverse protein profiles in CNS myeloid cells and CNS tissue from lipopolysaccharide- and vehicle-injected APPSWE/PS1ΔE9 transgenic mice implicate cathepsin Z in Alzheimer’s disease. *Front Cell Neurosci* 2018; 12: 397.

77. Tzeng T-C, Hasegawa Y, Iguchi R, et al. Inflammasome-derived cytokine IL18 suppresses amyloid-induced seizures in Alzheimer-prone mice. *Proc Natl Acad Sci U S A* 2018; 115: 9002–9007.

78. Wan L, Zhang Q, Luo H, et al. Codonopsis pilosula polysaccharide attenuates Aβ toxicity and cognitive defects in APP/PS1 mice. *Aging* 2020; 12: 13422–13436.

79. Wang X, Xia W, Li K, et al. Rapamycin regulates cholesterol biosynthesis and cytoplasmic ribosomal proteins in hippocampus and temporal lobe of APP/PS1 mouse. *J Neurol Sci* 2019; 399: 125–139.

80. Wang L, Zhang Y, Zhao Y, et al. Deep cervical lymph node ligation aggravates AD-like pathology of APP/PS1 mice. *Brain Pathol* 2019; 29: 176–192.

81. Wang C, Shen D, Hu Y, et al. Selective targeting of class I HDAC reduces microglial inflammation in the entorhinal cortex of young APP/PS1 Mice*. Int J Mol Sci* 2023; 24: 4805.

82. Wei C, Zhang W, Zhou Q, et al. Mithramycin A alleviates cognitive deficits and reduces neuropathology in a transgenic mouse model of Alzheimer’s disease. *Neurochem Res* 2016; 41: 1924–1938.

83. Woo JA, Boggess T, Uhlar C, et al. RanBP9 at the intersection between cofilin and Aβ pathologies: rescue of neurodegenerative changes by RanBP9 reduction. *Cell Death Dis* 2015; 6: 1676.

84. Woo JA, Zhao X, Khan H, et al. Slingshot-Cofilin activation mediates mitochondrial and synaptic dysfunction via Aβ ligation to β1-integrin conformers. *Cell Death Differ* 2015; 22: 921–934.

85. Woodhouse A, Fernandez-Martos CM, Atkinson RAK, et al. Repeat propofol anesthesia does not exacerbate plaque deposition or synapse loss in APP/PS1 Alzheimer’s disease mice. *BMC Anesthesiol* 2018; 18: 47.

86. Wu D-D, Cheng J, Zheng Y-N, et al. Neuroplastin 65 deficiency reduces amyloid plaque formation and cognitive deficits in an Alzheimer’s disease mouse model. *Front Cell Neurosci* 2023; 5: 1129773.

87. Wu Q, Wang B, Li Q-F, et al. SRC-1 knockout exerts no effect on amyloid β deposition in APP/PS1 mice. *Front Aging Neurosci* 2020; 12: 145.

88. Zhang Y, Hu Y, Han Z, et al. Cattle encephalon glycoside and ignotin ameliorate palmitoylation of PSD-95 and enhance expression of synaptic proteins in the frontal cortex of a APPswe/PS1dE9 mouse model of Alzheimer’s disease. *J Alzheimers Dis* 2022; 88: 141–154.

89. Zhao N, Yan Q-W, Xia J, et al. Treadmill exercise attenuates Aβ-induced mitochondrial dysfunction and enhances mitophagy activity in APP/PS1 transgenic mice. *Neurochem Res* 2020; 45: 1202–1214.

90. Bai B, Wang X, Li Y, et al. Deep multilayer brain proteomics identifies molecular networks in Alzheimer’s disease progression. *Neuron* 2020; 105: 975-991.e7.

91. Boeddrich A, Haenig C, Neuendorf N, et al. A proteomics analysis of 5xFAD mouse brain regions reveals the lysosome-associated protein Arl8b as a candidate biomarker for Alzheimer’s disease. *Genome Med* 2023; 15: 50.

92. Bundy JL, Vied C, Badger C, et al. Sex-biased hippocampal pathology in the 5XFAD mouse model of Alzheimer’s disease: A multi-omic analysis. *J Comp Neurol* 2019; 527: 462–475.

93. Chen P-C, Han X, Shaw TI, et al. Alzheimer’s disease-associated U1 snRNP splicing dysfunction causes neuronal hyperexcitability and cognitive impairment. *Nat Aging* 2022; 2: 923-940.

94. de Pins B, Cifuentes-Díaz C, Thamila Farah A, et al. Conditional BDNF delivery from astrocytes rescues memory deficits, spine density and synaptic properties in the 5xFAD mouse model of Alzheimer disease. *J Neurosci* 2019; 39: 2441–2458.

95. Henningfield CM, Arreola MA, Soni N, et al. Microglia-specific ApoE knock-out does not alter Alzheimer’s disease plaque pathogenesis or gene expression. *Glia* 2022; 70: 287–302.

96. Hurst CD, Dunn AR, Dammer EB, et al. Genetic background influences the 5XFAD Alzheimer’s disease mouse model brain proteome. *Front Aging Neurosci* 2023; 15: 1239116.

97. Jiang L-X, Huang G-D, Su F, et al. Vortioxetine administration attenuates cognitive and synaptic deficits in 5×FAD mice. *Psychopharmacology* 2020; 237: 1233–1243.

98. Ju IG, Kim N, Choi JG, et al. Cuscutae japonicae semen ameliorates memory dysfunction by rescuing synaptic damage in Alzheimer’s disease models. *Nutrients* 2019; 11: 2591.

99. Kang S, Ha S, H P, et al. Effects of a dehydroevodiamine-derivative on synaptic destabilization and memory impairment in the 5xFAD, Alzheimer’s disease mouse model. *Front Behav Neurosci* 2018; 12: 273.

100. Kim DK, Park J, Han D, et al. Molecular and functional signatures in a novel Alzheimer’s disease mouse model assessed by quantitative proteomics. *Mol Neurodegener* 2018; 13: 2.

101. Kim DK, Han D, Park J, et al. Deep proteome profiling of the hippocampus in the 5XFAD mouse model reveals biological process alterations and a novel biomarker of Alzheimer’s disease. *Exp Mol Med* 2019; 51: 1–17.

102. Kim J, Kang S, Chang K-A. Effect of cx-DHED on abnormal glucose transporter expression induced by AD pathologies in the 5xFAD mouse model. *Int J Mol Sci* 2022; 23: 10602.

103. Kiris I, Kukula-Koch W, Karayel-Basar M, et al. Proteomic alterations in the cerebellum and hippocampus in an Alzheimer’s disease mouse model: Alleviating effect of palmatine. *Biomed Pharmacother* 2023; 158: 114111.

104. Li H, Lai L, Li X, et al. Electroacupuncture ameliorates cognitive impairment by regulating γ-amino butyric acidergic interneurons in the hippocampus of 5 familial Alzheimer’s disease mice. *Neuromodulation* 2024; 27: 730–741.

105. Lopes FBTP, Schlatzer D, Wang R, et al. Temporal and sex-linked protein expression dynamics in a familial model of Alzheimer’s disease. *Mol Cell Proteomics* 2022; 21: 100280.

106. Na EJ, Jeon Y, Kim H, et al. Translationally controlled tumor protein restores impaired memory and altered synaptic protein expression in animal models of dementia. *Biomed Pharmacother* 2023; 160: 114357.

107. Neuner SM, Wilmott LA, Hoffmann BR, et al. Hippocampal proteomics defines pathways associated with memory decline and resilience in normal aging and Alzheimer’s disease mouse models. *Behav Brain Res* 2017; 322: 288–298.

108. Oh S-B, Kim MS, Park S, et al. Clusterin contributes to early stage of Alzheimer’s disease pathogenesis. *Brain Pathol* 2019; 29: 217–231.

109. Pérez-Sisqués L, Sancho-Balsells A, Solana-Balaguer J, et al. RTP801/REDD1 contributes to neuroinflammation severity and memory impairments in Alzheimer’s disease. *Cell Death Dis* 2021; 12: 616.

110. Son SM, Nam DW, Cha M-Y, et al. Thrombospondin-1 prevents amyloid beta–mediated synaptic pathology in Alzheimer’s disease. *Neurobiol Aging* 2015; 36: 3214–3227.

111. Sun F, Zhao J, Zhang H, et al. Proteomics evidence of the role of TDMQ20 in the cholinergic system and synaptic transmission in a mouse model of Alzheimer’s disease. *ACS Chem Neurosci* 2022; 13: 3093--3107.

112. Sweetat S, Nitzan K, Suissa N, et al. The beneficial effect of mitochondrial transfer therapy in 5XFAD mice via liver-serum-brain response. *Cells* 2023; 12: 1006.

113. Thanou E, Koopmans F, Pita-Illobre D, et al. Suspension TRAPping Filter (sTRAP) sample preparation for quantitative proteomics in the low µg input range using a plasmid DNA micro-spin column: Analysis of the hippocampus from the 5xFAD Alzheimer’s disease mouse model. *Cells* 2023; 12: 1242.

114. Tible M, Mouton Liger F, Schmitt J, et al. PKR knockout in the 5xFAD model of Alzheimer’s disease reveals beneficial effects on spatial memory and brain lesions. *Aging Cell* 2019; 18: e12887.

115. Vasilopoulou F, Rodríguez-Arévalo S, Bagán A, et al. Disease-modifying treatment with I2 imidazoline receptor ligand LSL60101 in an Alzheimer’s disease mouse model: A comparative study with donepezil. *Br J Pharmacol* 2021; 178: 3017–3033.

116. Wang W, Li Y, Ma F, et al. Microglial repopulation reverses cognitive and synaptic deficits in an Alzheimer’s disease model by restoring BDNF signaling. *Brain Behav Immun* 2023; 113: 275-288.

117. Wang E, Pan AL, Bagchi P, et al. Proteomic signaling of dual-specificity phosphatase 4 (DUSP4) in Alzheimer’s disease. *Biomolecules* 2024; 14: 66.

118. Yan L, Jin Y, Pan J, et al. 7,8-dihydroxycoumarin alleviates synaptic loss by activated PI3K-Akt-CREB-BDNF signaling in Alzheimer’s disease model mice. *J Agric Food Chem* 2022; 70: 7130–7138.

119. Yang E-J, Ahn S, Ryu J, et al. Phloroglucinol attenuates the cognitive deficits of the 5XFAD mouse model of Alzheimer’s disease. *PLoS One* 2015; 10: e0135686.

120. Babic Perhoc A, Osmanovic Barilar J, Knezovic A, et al. Cognitive, behavioral and metabolic effects of oral galactose treatment in the transgenic Tg2576 mice. *Neuropharmacology* 2019; 148: 50–67.

121. Do Carmo S, Crynen G, Paradis T, et al. Hippocampal proteomic analysis reveals distinct pathway deregulation profiles at early and late stages in a rat model of Alzheimer’s-like amyloid pathology. *Mol Neurobiol* 2018; 55: 3451-3476.

122. Hall H, Iulita MF, Gubert P, et al. AF710B, an M1/sigma-1 receptor agonist with long-lasting disease-modifying properties in a transgenic rat model of Alzheimer’s disease. *Alzheimers Dement* 2018; 14: 811–823.

123. Lipton SA, Rezaie T, Nutter A, et al. Therapeutic advantage of pro-electrophilic drugs to activate the Nrf2/ARE pathway in Alzheimer’s disease models. *Cell Death Dis* 2016; 7: e2499.

124. Lim SL, Tran DN, Kieu Z, et al. Genetic ablation of hematopoietic cell kinase accelerates Alzheimer’s disease-like neuropathology in Tg2576 mice. *Mol Neurobiol* 2020; 57: 2447–2460.

125. Palomino-Alonso M, Lachén-Montes M, González-Morales A, et al. Network-driven proteogenomics unveils an aging-related imbalance in the olfactory IκBα-NFκB p65 complex functionality in Tg2576 Alzheimer’s disease mouse model. *Int J Mol Sci* 2017; 18: 2260.

126. Ramos-Miguel A, Hercher C, Beasley CL, et al. Loss of Munc18-1 long splice variant in GABAergic terminals is associated with cognitive decline and increased risk of dementia in a community sample. *Mol Neurodegener* 2015; 10: 65.

127. Rodríguez Cruz Y, Strehaiano M, Rodríguez Obaya T, et al. An intranasal formulation of erythropoietin (Neuro-EPO) prevents memory deficits and amyloid toxicity in the APP Swe transgenic mouse model of Alzheimer’s disease. *J Alzheimers Dis* 2017; 55: 231–248.

128. Rofo F, Sandbaumhüter FA, Chourlia A, et al. Wide-ranging effects on the brain proteome in a transgenic mouse model of Alzheimer’s disease following treatment with a brain-targeting somatostatin peptide. *ACS Chem Neurosci* 2021; 12: 2529–2541.

129. Schnöder L, Quan W, Yu Y, et al. Deficiency of IKKβ in neurons ameliorates Alzheimer’s disease pathology in APP- and tau-transgenic mice. *FASEB J* 2023; 37: e22778.

130. Spencer B, Desplats PA, Overk CR, et al. Reducing endogenous α-synuclein mitigates the degeneration of selective neuronal populations in an Alzheimer’s disease transgenic mouse model. *J Neurosci* 2016; 36: 7971–7984.

131. Xu J, de Winter F, Farrokhi C, et al. Neuregulin 1 improves cognitive deficits and neuropathology in an Alzheimer’s disease model. *Sci Rep* 2016; 6: 31692.

132. Aladeokin AC, Akiyama T, Kimura A, et al. Network-guided analysis of hippocampal proteome identifies novel proteins that colocalize with Aβ in a mice model of early-stage Alzheimer’s disease. *Neurobiol Dis* 2019; 132: 104603.

133. Schedin-Weiss S, Nilsson P, Sandebring-Matton A, et al. Proteomics time-course study of App knock-in mice reveals novel presymptomatic Aβ42-induced pathways to Alzheimer’s disease pathology. *J Alzheimers Dis* 2020; 75: 321–335.

134. Medina-Vera D, Enache D, Tambaro S, et al. Translational potential of synaptic alterations in Alzheimer’s disease patients and amyloid precursor protein knock-in mice. *Brain Commun* 2023; 5: fcad001.

135. Ito S, Yagi R, Ogata S, et al. Proteomic alterations in the brain and blood-brain barrier during brain Aβ accumulation in an APP knock-in mouse model of Alzheimer’s disease. *Fluids Barriers CNS* 2023; 20: 66.

136. Apicco DJ, Ash PEA, Maziuk B, et al. Reducing the RNA binding protein TIA1 protects against tau-mediated neurodegeneration in vivo. *Nat Neurosci* 2018; 21: 72-80.

137. Hashem J, Hu M, Zhang J, et al. Inhibition of 2-arachidonoylglycerol metabolism alleviates neuropathology and improves cognitive function in a tau mouse model of Alzheimer’s disease. *Mol Neurobiol* 2021; 58: 4122–4133.

138. Soto-Faguás CM, Sanchez-Molina P, Saura CA. Loss of presenilin function enhances tau phosphorylation and aggregation in mice. *Acta Neuropathol Commun* 2021; 9: 162.

139. Sun X-Y, Dong Q-X, Zhu J, et al. Resveratrol rescues tau-induced cognitive deficits and neuropathology in a mouse model of tauopathy. *Curr Alzheimer Res* 2019; 16: 710–722.

140. Sun X-Y, Li L-J, Dong Q-X, et al. Rutin prevents tau pathology and neuroinflammation in a mouse model of Alzheimer’s disease. *J Neuroinflammation* 2021; 18: 131.

141. Schwab K, Melis V, Harrington CR, et al. Proteomic analysis of hydromethylthionine in the line 66 model of frontotemporal dementia demonstrates actions on tau-dependent and tau-independent networks. *Cells* 2021; 10: 2162.

142. Tang SJ, Fesharaki-Zadeh A, Takahashi H, et al. Fyn kinase inhibition reduces protein aggregation, increases synapse density and improves memory in transgenic and traumatic Tauopathy. *Acta Neuropathol Commun* 2020; 8: 96.

143. Zhong L, Sheng X, Wang W, et al. TREM2 receptor protects against complement-mediated synaptic loss by binding to complement C1q during neurodegeneration. *Immunity* 2023; 56: 1794-1808.e8.

144. Helboe L, Egebjerg J, Barkholt P, et al. Early depletion of CA1 neurons and late neurodegeneration in a mouse tauopathy model. *Brain Res* 2017; 1665: 22–35.

145. Jiao S-S, Shen L-L, Zhu C, et al. Brain-derived neurotrophic factor protects against tau-related neurodegeneration of Alzheimer’s disease. *Transl Psychiatry* 2016; 6: e907-e907.

146. Koren SA, Hamm MJ, Cloyd R, et al. Broad kinase inhibition mitigates early neuronal dysfunction in tauopathy. *Int J Mol Sci* 2021; 22: 1186.

147. Ren Q-G, Gong W-G, Zhou H, et al. Spatial training ameliorates long-term Alzheimer’s disease-like pathological deficits by reducing NLRP3 inflammasomes in PR5 mice. *Neurother* 2019; 16: 450–464.

148. Turnbull MT, Coulson EJ. Cholinergic basal forebrain lesion decreases neurotrophin signaling without affecting tau hyperphosphorylation in genetically susceptible mice. *J Alzheimers Dis* 2017; 55: 1141–1154.

149. Vijayan M, Alvir RV, Alvir RV, et al. A partial reduction of VDAC1 enhances mitophagy, autophagy, synaptic activities in a transgenic Tau mouse model. *Aging Cell* 2022; 21: e13663.

150. Wang Y-J, Gong W-G, Ren Q-G, et al. Escitalopram alleviates Alzheimer’s disease-type tau pathologies in the aged P301L tau transgenic mice. *J Alzheimers Dis* 2020; 77: 807–819.

151. Jadhav S, Katina S, Kovac A, et al. Truncated tau deregulates synaptic markers in rat model for human tauopathy. *Front Cell Neurosci* 2015; 9: 24.

152. Tiwari SS, d’Orange M, Troakes C, et al. Evidence that the presynaptic vesicle protein CSPalpha is a key player in synaptic degeneration and protection in Alzheimer’s disease. *Mol Brain* 2015; 8: 6.

153. Baazaoui N, Iqbal K. Prevention of dendritic and synaptic deficits and cognitive impairment with a neurotrophic compound. *Alzheimers Res Ther* 2017; 9: 45.

154. Broderick TL, Rasool S, Li R, et al. Neuroprotective effects of chronic resveratrol treatment and exercise training in the 3xTg-AD mouse model of Alzheimer’s disease. *Int J Mol Sci* 2020; 21: 7337.

155. Chen Y-J, Zheng H-Y, Huang X-X, et al. Neuroprotective effects of icariin on brain metabolism, mitochondrial functions, and cognition in triple-transgenic Alzheimer’s disease mice. *CNS Neurosci Ther* 2016; 22: 63–73.

156. Chen C, Jiang X, Li Y, et al. Low-dose oral copper treatment changes the hippocampal phosphoproteomic profile and perturbs mitochondrial function in a mouse model of Alzheimer’s disease. *Free Radic Biol Med* 2019; 135: 144–156.

157. Chen C, Liu P, Wang J, et al. Dauricine attenuates spatial memory impairment and Alzheimer-like pathologies by enhancing mitochondrial function in a mouse model of Alzheimer’s Disease. *Front Cell Dev Biol* 2021; 8: 624339.

158. Du X, Shi Q, Zhao Y, et al. Se-Methylselenocysteine (SMC) improves cognitive deficits by attenuating synaptic and metabolic abnormalities in Alzheimer’s mice model: A proteomic study. *ACS Chem Neurosci* 2021; 12: 1112-1132.

159. He Z, Zheng L, Zhao X, et al. An adequate supply of Bis(ethylmaltolato)oxidovanadium(IV) remarkably reversed the pathological hallmarks of Alzheimer’s disease in triple-transgenic middle-aged mice. *Biol Trace Elem Res* 2022; 200: 3248–3264.

160. Iqbal J, Zhang K, Jin N, et al. Effect of sodium selenate on hippocampal proteome of 3×Tg-AD mice-exploring the antioxidant dogma of selenium against Alzheimer’s disease. *ACS Chem Neurosci* 2018; 9: 1637–1651.

161. Kim D, Cho J and Kang H. Protective effect of exercise training against the progression of Alzheimer’s disease in 3xTg-AD mice. *Behav Brain Res* 2019; 374: 112105.

162. Kim T-W, Park S-S, Park J-Y, et al. Infusion of plasma from exercised mice ameliorates cognitive dysfunction by increasing hippocampal neuroplasticity and mitochondrial functions in 3xTg-AD mice. *Int J Mol Sci* 2020; 21: 3291.

163. Martínez-Drudis L, Sheta R, Pellegrinato R, et al. Inhibition of PLK2 activity affects APP and tau pathology and improves synaptic content in a sex-dependent manner in a 3xTg mouse model of Alzheimer’s disease. *Neurobiol Dis* 2022; 172: 105833.

164. Nie L, Xia J, Li H, et al. Ginsenoside Rg1 ameliorates behavioral abnormalities and modulates the hippocampal proteomic change in triple transgenic mice of Alzheimer’s disease. *Oxid Med Cell Longev* 2017; 2017: 6473506.

165. Nie L, Wei G, Peng S, et al. Melatonin ameliorates anxiety and depression-like behaviors and modulates proteomic changes in triple transgenic mice of Alzheimer’s disease. *BioFactors* 2017; 43: 593–611.

166. Nie L, He K, Xie F, et al. Loganin substantially ameliorates molecular deficits, pathologies and cognitive impairment in a mouse model of Alzheimer’s disease. *Aging* 2021; 13: 23739–23756.

167. Sun D-S, Gao L-F, Jin L, et al. Fluoxetine administration during adolescence attenuates cognitive and synaptic deficits in adult 3×TgAD mice. *Neuropharmacology* 2017; 126: 200-212.

168. Trujillo-Estrada L, Vanderklish PW, Nguyen MMT, et al. SPG302 reverses synaptic and cognitive deficits without altering amyloid or tau pathology in a transgenic model of Alzheimer’s disease. *Neurotherapeutics* 2021; 18: 2468–2483.

169. Vandal M, White PJ, Tournissac M, et al. Impaired thermoregulation and beneficial effects of thermoneutrality in the 3×Tg-AD model of Alzheimer’s disease. *Neurobiol Aging* 2016; 43: 47–57.

170. Wang H, Zhang Z, Hongpaisan J. PKCε activator protects hippocampal microvascular disruption and memory defect in 3×Tg-Alzheimer’s disease mice with cerebral microinfarcts. *Front Aging Neurosci* 2023; 15: 1272361.

171. Wei W, Liu Y, Dai C-L, et al. Neurotrophic treatment initiated during early postnatal development prevents the alzheimer-like behavior and synaptic dysfunction. *J Alzheimers Dis* 2021; 82: 631–646.

172. Yagensky O, Kohansal-Nodehi M, Gunaseelan S, et al. Increased expression of heme-binding protein 1 early in Alzheimer’s disease is linked to neurotoxicity. *Elife* 2019; 8: e47498.

173. Ying M, Sui X, Zhang Y, et al. Identification of novel key molecules involved in spatial memory impairment in triple transgenic mice of Alzheimer’s disease. *Mol Neurobiol* 2017; 54: 3843-3858.

174. Yu J, Luo X, Xu H, et al. Identification of the key molecules involved in chronic copper exposure-aggravated memory impairment in transgenic mice of Alzheimer’s disease using proteomic analysis. *J Alzheimers Dis* 2015; 44: 455-469.

175. Yu Y-Z, Li Q-L, Wang H-C, et al. Improved synaptic and cognitive function in aged 3 × Tg-AD mice with reduced amyloid-β after immunotherapy with a novel recombinant 6Aβ15-TF chimeric vaccine. *Clin Immunol* 2018; 193: 12–23.

176. Yue C, Shan Z, Tan Y, et al. His-rich domain of selenoprotein P ameliorates neuropathology and cognitive deficits by regulating TrkB pathway and zinc homeostasis in an Alzheimer model of mice. *ACS Chem Neurosci* 2020; 11: 4098–4110.

177. Zhou X, Wang L, Xiao W, et al. Memantine improves cognitive function and alters hippocampal and cortical proteome in triple transgenic mouse model of Alzheimer’s disease. *Exp Neurobiol* 2019; 28: 390-403.

178. Zhou X, Xiao W, Su Z, et al. Hippocampal proteomic alteration in triple transgenic mouse model of Alzheimer’s disease and implication of PINK 1 regulation in donepezil treatment. *J Proteome Res* 2019; 18: 1542–1552.

179. Zhou Y, Yang D, Chen H, et al. Polyphyllin I attenuates cognitive impairments and reduces AD-like pathology through CIP2A-PP2A signaling pathway in 3XTg-AD mice. *FASEB J* 2020; 34: 16414–16431.

180. Zhou X, Huang K, Wang Y, et al. Evaluation of therapeutic effects of tetramethylpyrazine nitrone in Alzheimer’s disease mouse model and proteomics analysis. *Front Pharmacol* 2023; 14: 1082602.

181. Chhibber A, Zhao L. ERβ and ApoE isoforms interact to regulate BDNF-5-HT2A signaling and synaptic function in the female brain. *Alzheimers Res Ther* 2017; 9: 79.

182. Yin J, Nielsen M, Li S, et al. Ketones improves Apolipoprotein E4-related memory deficiency via sirtuin 3. *Aging* 2019; 11: 4579–4586.

183. Yin J, Nielsen M, Carcione T, et al. Apolipoprotein E regulates mitochondrial function through the PGC-1α-sirtuin 3 pathway. *Aging* 2019; 11: 11148–11156.

184. Pont C, Ginex T, Griñán-Ferré C, et al. From virtual screening hits targeting a cryptic pocket in BACE-1 to a nontoxic brain permeable multitarget anti-Alzheimer lead with disease-modifying and cognition-enhancing effects. *Eur J Med Chem* 2021; 225: 113779.
